# Neuroscience-based relational art therapy and deep brain reorienting in the treatment of dissociative identity disorder

**DOI:** 10.3389/fpsyg.2025.1454483

**Published:** 2025-02-28

**Authors:** Anna Gerge, Gabriella Rudstam, Hans Peter Söndergaard

**Affiliations:** ^1^Department of Communication and Psychology, Aalborg University, Aalborg, Denmark; ^2^Department of Clinical Neuroscience, Karolinska Institutet, Stockholm, Sweden

**Keywords:** attachment trauma, dissociative identity disorder (DID), states of being, relational art therapy (RAT), deep brain reorienting (DBR), brain physiology, functional networks, change

## Abstract

Art therapy (AT) has been proposed as a treatment for post-traumatic conditions, potentially by providing somatic sensory input that can (i) enhance the client’s sense of self and embodiment, (ii) modulate arousal, and (iii) aid in rethinking and reframing traumatic memories. However, evidence supporting AT as a treatment for dissociative disorders remains limited. The theoretical basis for the efficacy of AT is discussed in relation to findings regarding the traumatized person’s brain and mindset, as well as its altered functional network connectivity. It is crucial to consider specific alterations in brain networks associated with trauma, particularly those occurring in the deep brain regions, which include the midbrain, the brainstem, and the cerebellum. The hypothesis suggests that early or severe trauma can impair the brain’s higher regulatory functions, as explained by the cascade theory. This theory explains how diverse activation patterns within the midbrain’s periaqueductal gray (PAG) of the midbrain influence the limbic system and cortices, thereby modulating states of being and behavior. Phase-specific, resource-oriented, and long-term therapy for complexly traumatized and dissociative individuals can benefit from novel insights from neuroimaging studies to inform and enhance therapeutic methods. This is illustrated in a clinical vignette with a client diagnosed with dissociative identity disorder (DID), where deep brain reorienting (DBR) was combined with relational AT. The AT component is hypothesized to have facilitated a sense of grounding in the present moment and enhanced the client’s access to her neurophenomenological self. Moreover, changes may have occurred at implicit and non-verbal levels. DBR is believed to have helped the client remain present with her previously avoided and unbearable internal experience. To validate these assumptions, the second author conducted a semi-structured interview that focused on the client’s experiences of *being dissociative and in psychotherapy*, including the effect of DBR when introduced after AT. The client’s experiences were articulated through a thematic analysis of the interview, which yielded the following themes: *Loneliness, getting help*, and *moving towards togetherness*. Further research on and development of therapy methods that enhance the neuroplasticity necessary for highly dissociative clients to change and heal are highly recommended.

## Introduction

The article offers a review of research on how our minds and brains are affected by early traumatization. The subsequent section delineates the therapeutic interventions necessary to facilitate the transition of clients with early trauma, who often struggle with intrusive memories and depersonalization, toward a sense of agency and control over their past experiences. A clinical vignette illustrates this process using a client with dissociative identity disorder (DID). DID is characterized as a trauma-induced condition exhibiting discontinuities in the sense of self and agency (DSM 5-TR, 2022). The clinical vignette will show how RAT and DBR can be applied during therapy, positive changes supposedly related to the therapy, and the client’s reflections on the experience after/during therapy. AT is a therapeutic modality that utilizes creative visual techniques to facilitate individuals’ self-expression to examine their nonverbal arts-based creations, encompassing symbols and metaphors (King, 2016). The relational art-making process in RAT is grounded in relationally attuned, psychodynamic interventions (Springham and Huet, 2018). Both AT and RAT are theorized to enhance self-understanding, agency, and the individual’s capacity to relate to and overcome limitations and challenges (King, 2016). DBR is a trauma psychotherapy that aims to facilitate the processing of traumatic experiences by tracking the original sequence of physiological responses that occurred when parts of the brain’s deep structures, e.g., the brainstem and the midbrain, were alerted to a threat or an attachment disruption (Corrigan and Christie-Sands, 2020; Corrigan et al., 2025). The objectives of this article are to:

A, give an overview of recent neurobiological findings concerning the impact of traumatization, including attachment wounds and neglect, on the developing brain and this subsequent influence on the integrative capacity of adult clients, as shown by studies of connectivity by fMRI.

B, sketch a framework for the relational and neurophysiological processes that affect AT-based interventions with severely traumatized and dissociative clients.

C, underscore the vulnerability of appraisal processes in such clients and emphasize the necessity for interventions, including arts-based ones and DBR, to be adapted to accommodate this instability.

D, discuss the potential of RAT and DBR, when utilized in conjunction, to amplify healing processes, particularly in cases of severe dissociative disorders, such as DID.

The reflections should be meaningful for other therapists who employ somatic sensory input methods with clients struggling with the after-effects of significant traumatization, as outlined by Kearney and Lanius (2022). These methods have the capacity to modulate arousal, thereby facilitating clients’ progression towards a coherent sense of self. This assertion is supported by empirical evidence from expressive arts therapies, which include the therapeutic application of music, dance, theatre, art, and creative writing in therapeutic settings (Baker et al., 2017; Hass-Cohen and Clyde Findlay, 2015; King, 2016; King et al., 2019; Malchiodi, 2020; Rudstam, 2023; Vaisvaser et al., 2024). The capacity to modulate arousal also applies to DBR, a novel approach to the treatment of trauma-related disorders (Corrigan and Christie-Sands, 2020; Corrigan et al., 2025; Kearney et al., 2023a). DBR aims to access the core of a traumatic experience by tracking the original physiological sequence in the midbrain and brainstem.

In therapeutic practice, several therapy methods might be used simultaneously. Expressive arts imply a multimodal approach (Levine and Levine, 2011; Malchiodi, 2020; Rappaport, 2022; Vaisvaser et al., 2024). Art therapy (AT) can be a multi-faceted phenomenon due to clinical adaptations to certain client groups and the therapist’s orientation. Guided Imagery and Music (GIM) interventions have been found to incorporate AT (Rudstam et al., 2022). The development of mixed forms of AT and mindfulness has emerged from a combination of Gendlin’s Focusing (Gendlin, 1978) and AT, namely, focusing-oriented art therapy (FOAT) (Rappaport, 1988, 2010) or AT and mindfulness-based stress reduction (MBSR) (Joshi et al., 2021). Cognitive behavioral therapy (CBT) (Sarid et al., 2017), psychodynamic approaches (Eren et al., 2014), and person-centered approaches of AT (Rappaport, 2022) have been developed and researched. AT has been integrated (Gomez, 2019; Sigal and Rob, 2021) in eye movement desensitization and reprocessing (EMDR) (Shapiro, 1995, 2012). AT is a therapeutic technique that aims to bypass the spoken word and engage the sensing body. This process is believed to activate neuronal circuits that reshape behavior, images, emotions, and cognitions (Malchiodi, 2020; Schore, 2012; Vaisvaser, 2021).

### Interpersonal factors within therapy and art therapy

In their seminal work on the effect sizes of psychotherapy, Wampold and Imel (2015) found that any psychotherapy was more effective than no treatment (*d* = 0.80). The average differences in efficacy between specific practices were small, *d* < 0.20. Common factors, such as alliance (*d* = 0.57), empathy (*d* = 0.63), and congruence (*d* = 0.49), were found to have a substantial impact on the outcomes of the studied therapies. In the context of AT, the therapeutic relationship can be regarded as reinforced and solidified during the art-making process (Vaisvaser et al., 2024), thereby amplifying these common factors contingent upon the client’s acceptance of the treatment modality. Other methods can also promote positive outcomes through their specific interventions.

In the context of therapy, the development of an earned secure attachment (Schore, 2003a,b) can be facilitated by poignant “moments of meeting” (Duarte et al., 2020; Stern, 2004). Consequently, clients’ “implicit relational knowing” (Boston Change Process Study Group, 2010, 2018; Stern et al., 1998) can undergo transformation. This transformation is purportedly facilitated by moments of interpersonal neuronal synchrony (Redcay and Schilbach, 2019), where collaborative attention of therapist and client in RAT can foster new embodied narratives (Springham and Huet, 2018). In conjunction with art as a “third hand,” the beneficial, non-intrusive support provided by an attuned therapist can potentially enhance novel experiences of togetherness (Carr, 2014; Kramer, 1972). Such experiences allow for changes in the implicit relational domain and potentially rebuild attachment patterns through shared primary intersubjectivity (Trevarthen, 1979). In this way, the RAT experience shares similarities with the early developing sensory-motor processes of interaction between infant and caregiver. The efficacy of RAT may be partly attributed to its ability to concretize the therapist’s care.

The therapeutic relationship is potentially rendered more authentic through engagement in relational aesthetic experiences (Vaisvaser et al., 2024), facilitating the expression, experience, and sharing of positive effects and emotional states. Such an approach has the potential to alleviate affect phobia (Nijenhuis, 2017), foster the development of new states of being, and lead to altered regulation patterns. The “art as co-therapist” approach has been shown to facilitate experiences that can lead to changes in deep perceptions of self-others. Vaisvaser et al. (2024) postulated that the integration of sensory perception and salience detection with self-referential processing is a plausible hypothesis for the efficacy of AT. Consequently, heightened levels of (epistemic) trust, as posited by Fonagy and Luyten (2015) and Li et al. (2023), can be attained.

A drawing of one’s body can be defined as the appraised sum of the neurophenomenological self’s experience of the lived body from a first-person perspective (Lutz et al., 2024) and its images of itself in the world at a given time (Gerge et al., 2020). Body images can deepen clients’ understanding of implicit processes and increase their regulative capacity. Recent advancements in the field of neuroscience have unveiled that the sense of self and the sense of others are intricately linked to the brain’s ability to effectively integrate and segregate multiple networks (Di Plinio et al., 2020). These networks are influenced by external stimuli, such as artistic experiences (Bolwerk et al., 2014; Hutton, 2014; Vessel et al., 2013). The potential of the arts to facilitate integration in therapy is an area of research still in its early stages of development (Carolan and Stafford, 2018; Malhotra et al., 2024). In the context of interpersonal relationships, the arts have been shown to have a positive impact on sensory experiences and related brain activity (Vaisvaser et al., 2024).

## The trauma spectrum

Post-traumatic stress disorder (PTSD) is a psychiatric disorder that afflicts people who have experienced or witnessed traumatic events. The condition is characterized by intrusive memories, avoidance behaviors, and alterations in cognitive and emotional processes (DSM 5-TR, 2022). A systematic review of recently published observational studies (2015–2019) on PTSD in the US revealed significant variation in prevalence estimates due to differences in study designs (Schein et al., 2021). The 1-year prevalence ranged from a minimum of 2.3% to a maximum of 9.1%, and the lifetime prevalence from 3.4 to 26.9%. In military populations, the 1-year prevalence varied from 6.7 to 50.2%, and the lifetime prevalence from 7.7 to 17.0%. PTSD is frequently accompanied by other conditions, including dissociative symptoms, which affect up to 44% of individuals with the disorder. Additional symptoms of derealization and depersonalization are characteristic of the condition (Lanius et al., 2010a; White et al., 2022).

Complex PTSD (CPTSD) is prevalent in both clinical and general population samples, with a 1–8% population prevalence and up to 50% prevalence in mental health facilities (Maercker et al., 2022). In clinical samples of trauma victims, preliminary evidence suggests that CPTSD is a more prevalent condition than PTSD (Karatzias et al., 2018). Complex PTSD is regarded as a more severe form with changes in self-organization and more pronounced personality changes compared to PTSD. CPTSD is associated with traumatic events that begin earlier in life and are perpetrated by acquaintances (Guzman Torres et al., 2023). Not only childhood trauma but also other forms of inescapable stress in adulthood, such as vehement and overwhelming experiences fulfilling the requirements of A-criteria, are associated with PTSD and CPTSD. A UK study of asylum seekers and victims of trafficking revealed that two-thirds (66.23%) met the criteria for ICD-11 CPTSD (Jowett et al., 2021). The symptoms of CPTSD include those of PTSD, as well as disturbances in self-organization (DSO). These disturbances include affective dysregulation, characterized by severe and persistent challenges in managing emotions; a negative self-concept and low self-worth; and difficulties in relationships, which manifest as struggles in feeling close to others and maintaining interpersonal relationships (ICD-11, 2022).

The impact of traumatization on the body, mind, and brain is well understood by clinicians and traumatized individuals alike. In the following discussion, disorders from the trauma spectrum will be examined, including posttraumatic stress disorder (PTSD), complex posttraumatic stress disorder (CPTSD), PTSD of a dissociative subtype, and DID. This is because, in most cases, individuals with DID exhibit symptoms consistent with all the mentioned syndromes (Loewenstein et al., 2024). However, more severe and earlier-onset abuse appears to distinguish DID from other disorders (Boon and Draijer, 1993). Severe, chronic childhood trauma was found to be present in the histories of almost all individuals with DID (Dorahy et al., 2014), and Raison and Andrea (2023) found in their systematic review of data from 1990 to 2022 that DID appears to be more correlated to reported childhood traumas than other disorders. However, the researchers state that more research is required because DID remains understudied (Raison and Andrea, 2023).

The toxic level of stress resulting from early and ongoing exposure to adverse childhood experiences (ACEs) (Anda et al., 2006; Novais et al., 2021). has been shown to increase the risk of developing complex refractory posttraumatic conditions, including severe pathological dissociation (Schalinski et al., 2016; Teicher et al., 2022; Loewenstein et al., 2024). According to Teicher and Samson (2016), the neurobiological effects of abuse and neglect on the developing brain may result in a persistent sense of fear, heightened stress responses, and challenges in maintaining calm. Various forms of childhood trauma have been shown to impact the central nervous system, its functional networks, and the brain’s neuroanatomy, contributing to significant difficulties in self-regulation, agency, and interpersonal relations (Teicher and Samson, 2016). This phenomenon is believed to be influenced by elevated stress hormone levels during critical periods of development, particularly in cases where the child is subject to the influence of malevolent significant others. The absence of experiences essential for the development of the social brain in early childhood, as determined by genetic predispositions, can also have a detrimental impact on brain development and personality formation (Schore, 2009). Medicated depression is a condition in which childhood trauma has been found to be remarkably high, with a magnitude of 75.6% (Negele et al., 2015). Moreover, borderline personality disorder (BPD) is a syndrome where researchers highlighted the trauma-generated antecedents of the condition (Bozzatello et al., 2021; Lieb et al., 2004; van der Kolk et al., 1994). Greater attention is asked for regarding the relationship between BPD and DID, as BPD dissociation was associated with increased BPD symptom severity, self-harm, and reduced psychotherapy treatment response (Al-Shamali et al., 2022). According to Ross (2000), the absence of adequate love, care, and protection in early childhood can have devastating consequences. Dissociative and overmodulated subtypes of PTSD, such as the PTSD dissociative subtype, have been associated with a higher risk of depression and more childhood adversities in comparison to PTSD (Lanius et al., 2012; Schalinski et al., 2016; van Huijstee and Vermetten, 2018). PTSD of a dissociative subtype encompasses symptoms such as feelings of detachment and disruption in one’s sense of self (depersonalization) and surroundings (derealization). This phenomenon is associated with an elevated risk of suicidal self-injury (Srinivasan et al., 2022). From this perspective, PTSD is neither the sole nor the most prevalent condition experienced by survivors in the aftermath of trauma (Grossman et al., 2017).

### Dissociative identity disorder

DID affects 1–1.5% of the general population (Tyson and Brand, 2017; Loewenstein et al., 2024). Foote et al. (2006) found that dissociative disorders (DD) were highly prevalent (29%) in a clinical population with high prevalence rates for childhood physical and sexual abuse. In chronic outpatients requiring long-term care, 6% of subjects met the diagnostic criteria for DID (Foote et al., 2006), and typically, the condition had not been previously diagnosed. DD is thus prevalent in clinical populations, albeit underdiagnosed (Brand et al., 2016; Hawayek, 2023). In several outpatient studies, 12–29% of subjects met the criteria for DD, while 2–6% met the criteria for DID, with an average of 5% (Loewenstein et al., 2024).

Trauma-related pathological dissociation is characterized by disruptions in one’s sense of self, perceptions, and affective responses (Lebois et al., 2022, 2023). The predisposition for developing DID (Dorahy et al., 2014, 2015) reportedly includes an innate high capacity to enter hypnotic states and/or to dissociate (Dell, 2009; Kluft, 2009, 2012; Purcell et al., 2024). The development of DID is believed to be influenced by early childhood trauma, with the condition arising from the interaction of a child’s early experiences with caregivers who exhibit a lack of emotional regulation or who are perceived as frightening or abusive (Loewenstein et al., 2024). The hypothesis suggests that experiences of early childhood trauma can lead to a disruption in the development of interpersonal regulation, potentially resulting in the manifestation of DID. However, the role of D-attachment in this process remains a subject of debate (Granqvist et al., 2017), though the presence of D-attachment has been documented (Lyons-Ruth et al., 2006). Children with type-D attachment and betrayal trauma scripts (Herman, 2011) may be more susceptible to dissociative development (Loewenstein, 2023), which is hypothesized to be related to severe shame experiences when seeking attachment and comfort from unpredictable caregivers (Loewenstein, 2023).

The phenomenon of DID has been shown to be a syndrome with distinct neurobiological markers, according to Lebois et al. (2023), Purcell et al. (2024), and Lebois et al. (2021), and a coping mechanism when a child endures severe interpersonal dysregulation (Loewenstein, 2023). DID has been described as a phenomenologically distinct way of being (Reinders et al., 2014; Lebois et al., 2022). DID is theorized to be a response to an interpersonal reality, particularly in cases where caregivers have been unhelpful or even threatening during early childhood (Liotti, 2013; van der Hart, 2018). DID is characterized by chronically evoked defense states, marked by helplessness, hypo-arousal, and lower levels of consciousness (Loewenstein et al., 2024). In DID, these states can be interrupted by hyper-aroused states (Lebois et al., 2022). According to Purcell et al. (2024), DID exists on a continuum with PTSD, with more severe dissociative symptoms corresponding to a more dominant internally oriented and goal-directed cognition (Purcell et al., 2024). This is, supposedly, an early-developed strategy to rely on internal problem-solving in the face of unreliable or dangerous caregivers. In such upbringings, the capacity to disengage from sensory experiences might be advantageous in preventing overwhelming distress. Such adaptations may have been advantageous in the past, but they can impede the future lives of individuals with severe dissociative tendencies.

### The brain structures and functions post-traumatization

The subsequent section will delineate the way the human nervous system may be influenced by psychological trauma and severe attachment wounds. At present, the causality of these findings necessitates further elaboration, as the novel neurobiological findings are still in their nascent stages, and the field is undergoing rapid development. However, upon ongoing validation, these findings have the potential to substantially elucidate and enhance psychotherapeutic interventions, including an understanding of nonverbal change processes. Dysregulation and post-traumatic symptoms may arise when threats and attachment distortions affect deep brain regions, such as the brainstem, the midbrain, and the cerebellum (Perry, 2019; Perry et al., 2020; Kearney and Lanius, 2022). This, in turn, can disrupt the cortices, potentially resulting in a deficiency in integration between the brain’s evolutionary psychobiological systems. Consequently, the brain’s nested hierarchies may no longer function optimally (Panksepp, 1998, 2012). The prefrontal cortex (PFC) and the hippocampus are important in top-down regulation and amplifying signals and network dynamics underlying exploratory behavior (Malik et al., 2022). They are in contact with the left and right amygdala in the brain’s temporal lobes. The amygdalae regulate various functions, including impulsivity, aggressivity, fear learning, and fear extinction (Hamm and Weike, 2005).

However, in cases of complex traumatization, these structures can be compromised. The auditory, visual, and somatosensory cortices, along with the pathways in the midbrain that process and convey aversive experiences, are particularly vulnerable, according to Teicher and Samson (2016). Then, aversive experiences can lead to enduring changes in brain function and HPA axis responsiveness to stress (Teicher and Samson, 2016), associated with morphological alterations in the dorsal lateral prefrontal cortex (dlPFC), insula, and precuneus (Zhong et al., 2020). A constant state of hyperarousal in response to perceived stressful situations can lead to changes in the size of brain structures and how they interact with each other, according to Chalavi et al. (2015a,b) and McEwen et al. (2016). The presence of structural and functional alterations in the brain has been observed in individuals with PTSD (Hedges and Woon, 2010; Karl and Werner, 2010), PTSD of a dissociative subtype (Lotfinia et al., 2020), CPTSD (Schlumpf et al., 2022), and DID (Purcell et al., 2024).

A comprehensive review of magnetic resonance imaging (MRI) findings on structural and functional alterations in the brain of psychiatric patients revealed that brain structures such as the frontal and temporal cortices, amygdalae, hippocampus, insulae, and the brainstem appear to be impacted by traumatic experiences, particularly in patients with dissociative symptoms (Lotfinia et al., 2020). For instance, patients with DID exhibited smaller cortical and subcortical volumes in the hippocampus and amygdala when structural volume was studied with MRI. Additionally, parietal structures implicated in perception and personal awareness exhibited diminished volumes, as did frontal structures involved in movement execution and fear learning, according to Blihar et al. (2020).

Another comprehensive study revealed that individuals with PTSD exhibited significant gray and white matter reductions in the cerebellum compared to controls. The persons with PTSD demonstrated smaller total cerebellar volume and reduced volume in specific subregions compared to healthy controls (Huggins et al., 2024). Furthermore, cortical thinning was identified in women with PTSD following physical, emotional, and/or sexual assaults perpetrated by a spouse or intimate partner (Lee et al., 2023). Additionally, survivors of torture with PTSD exhibited cortical thinning compared to healthy individuals, according to Liddell et al. (2022). Prolonged stress has been demonstrated in several studies to result in a reduction of the hippocampus’s volume and functions at the cellular level (Almarzouki, 2024). Early life stress has been shown to alter hippocampal plasticity and memory (Kim et al., 2006). This phenomenon occurs independently of the diagnosis of PTSD, although additional hippocampal reduction has been observed in individuals with PTSD compared to a trauma-exposed group without PTSD (Woon et al., 2010).

**Figure 1:**
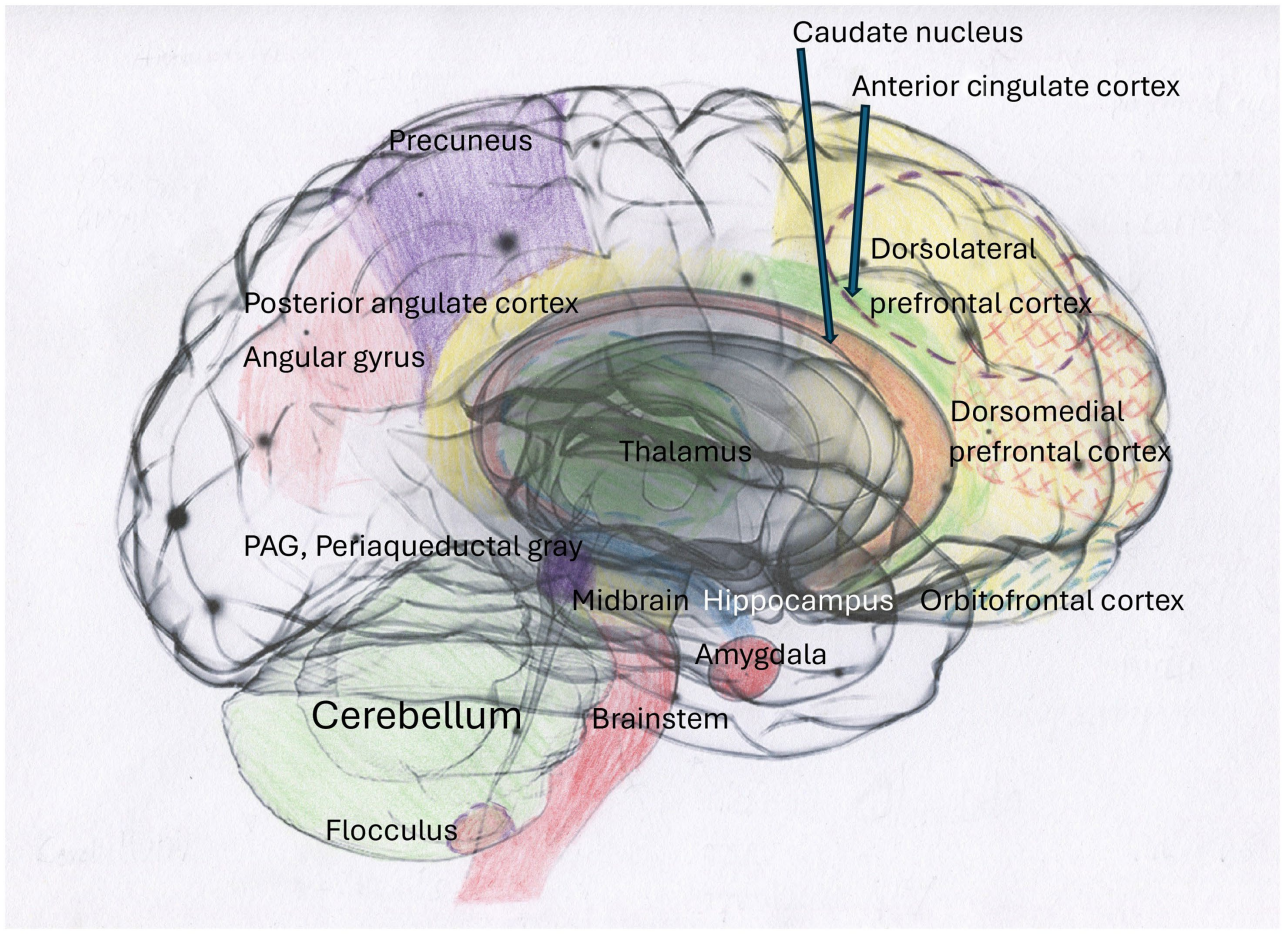
The brain exhibits structural alterations in response to traumatization, as evidenced by the extant literature. The impact of complex traumatization on the brain has been demonstrated in a wide range of studies, with research highlighting the involvement of the hippocampus in PTSD (Logue et al., 2018) and in cohorts with DID and other dissociative disorders (Chalavi et al., 2015a,b). Furthermore, brain scans have shown cortical thinning in torture survivors with PTSD (Lee et al., 2023; Liddell et al., 2022). Atrophy of the hippocampus and amygdala due to prolonged high stress has also been found (McEwen and Gianaros, 2010; Liddell et al., 2022). Furthermore, reduced amygdala volume, partly due to hypometabolism in the caudate nucleus compared to healthy controls, was also found in torture survivors (Liddell et al., 2022). In addition, people with PTSD also had smaller total cerebellar volume and reduced volume in subregions compared to healthy controls (Huggins et al., 2024).

**Figure 2:**
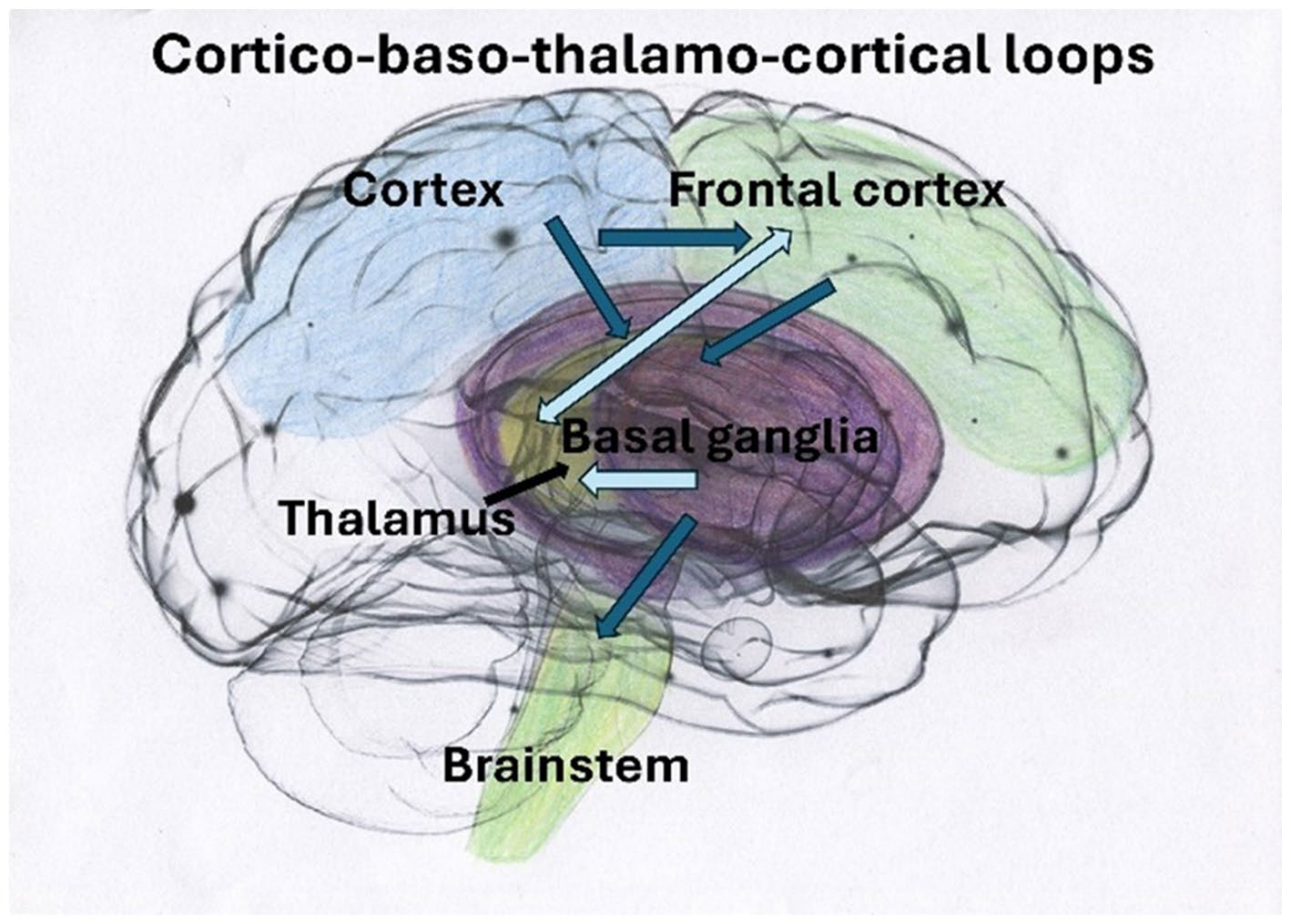
Traumatic experiences have a pervasive, cascading impact on both vertical and horizontal connectivity within the brain (Kearney and Lanius, 2022). These circuits of the SN play a crucial role in regulating behaviors. When these circuits become disrupted, they may contribute to visual fear processing through pathways from superior colliculi in the midbrain, impacting thalamus–amygdala pathways (Yoshii, 2021). This disruption can lead to symptoms such as rumination, obsessiveness, and a hollow sense of bodily self (Corrigan and Christie-Sands, 2020).

**Figure 3:**
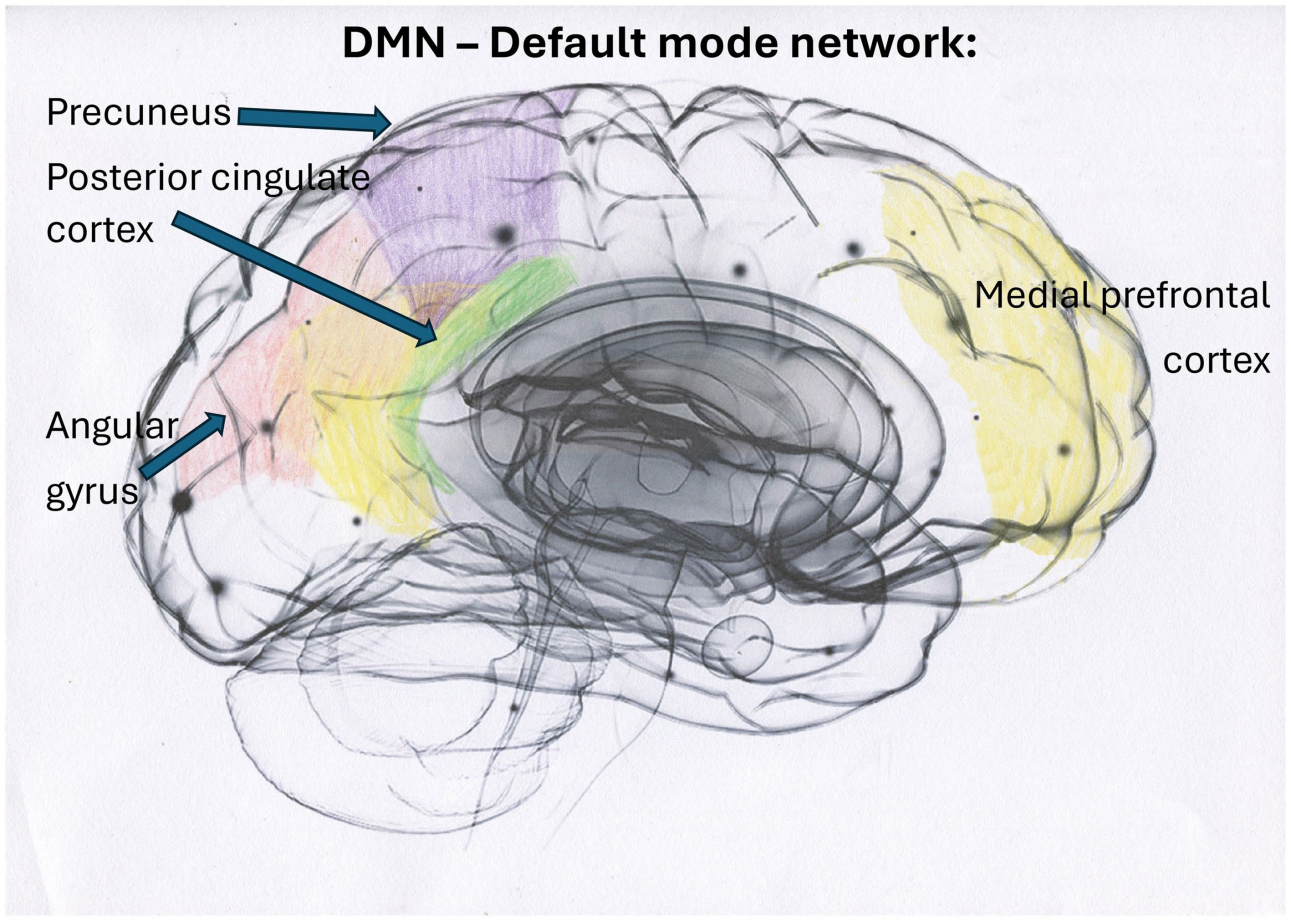
The DMN constitutes a large-scale brain network predominantly comprising cortical midline structures, including the dorsal, anterior, and ventral medial prefrontal cortex (MPFC), the posterior cingulate cortex (PCC), precuneus, the left and right parietal cortex, and the angular gyrus. In individuals who have not experienced trauma, the DMN facilitates spontaneous introspection and the capacity for time travel (Kearney and Lanius, 2024). The relationship between early trauma, disturbances in DMN regions, and psychopathology has been uniformly confirmed (Tian et al., 2022), and severe trauma impacts DMN functioning (Liddell et al., 2022). According to Liddell et al. (2022), torture survivors showed increased dynamic functional connectivity between the CEN and DMN, which suggests an adaptive over-regulative response with heightened top-down cognitive control. This response can result in diminished self-knowledge capacity and potentially trigger emotional rigidity, inflexibility, and withdrawal. The close connection between the DMN and deeper brain regions, such as the caudate nucleus, a region that is downregulated after torture, suggests that traumatized individuals may experience impaired procedural and associative learning, as well as reduced inhibitory control of actions (Liddell et al., 2022).

**Figure 4:**
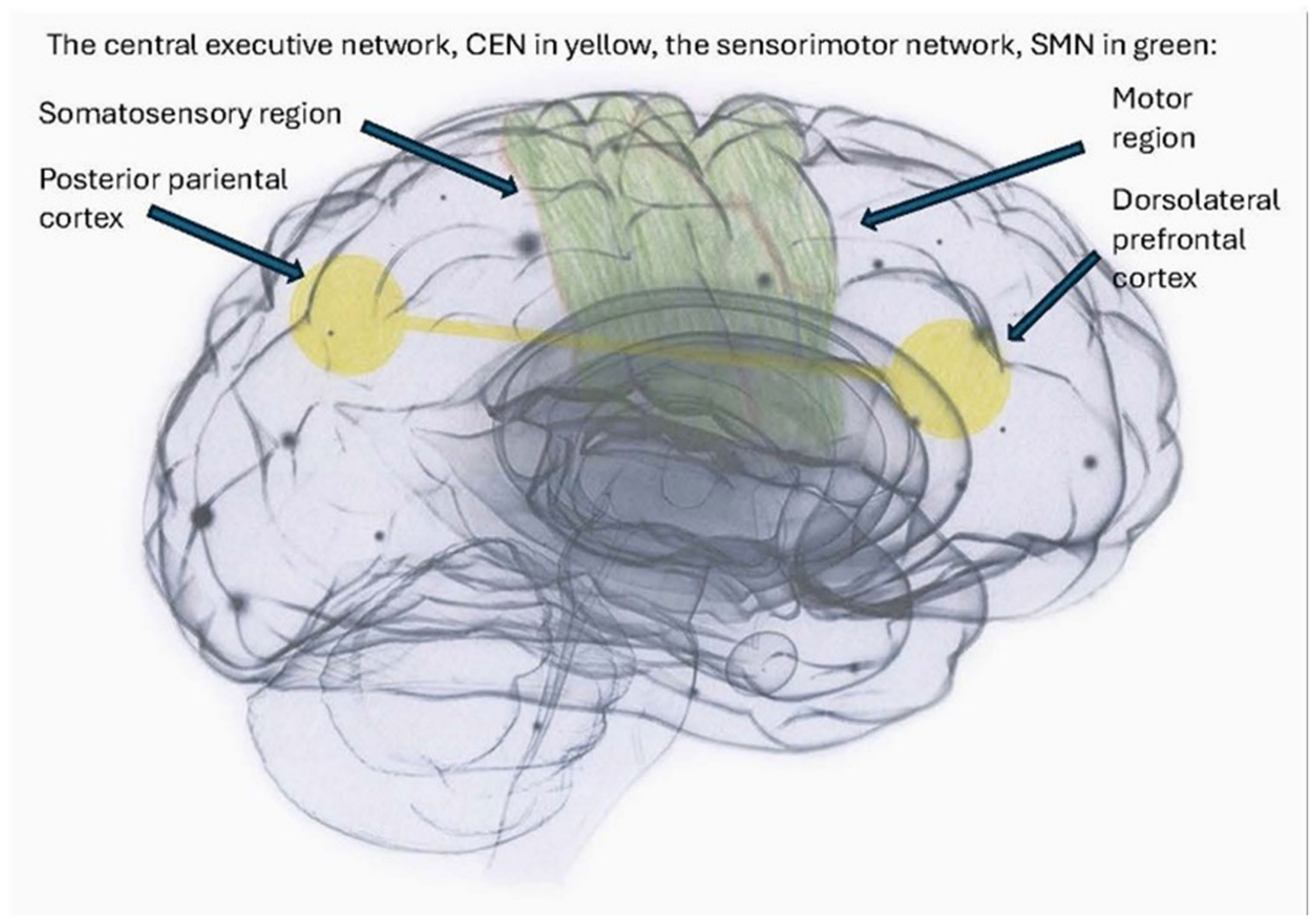
The sensorimotor network (SMN) and the central executive network (CEN) are implicated in executive function and goal-directed behavior in healthy subjects (Uddin et al., 2019). However, in complex traumatization, the CEN overmodulates subcortical brain activity, resulting in the suppression of somatic sensory information from deeper brain regions from reaching higher-order regions. Consequently, the sensorimotor network (SMN) and the posterior default mode network (DMN), as illustrated in Figure 3, are hypothesized to be hyper-coupled, resulting in diminished differentiation (Kearney et al., 2023b). Such a neurophysiological basis could contribute to the array of dissociative states frequently observed in complex traumatization and dissociation, including depersonalization, re-experiencing flashbacks, and decontextualized flashbacks.

Smaller hippocampal volumes have been reported in individuals with PTSD and DID, according to Chalavi et al. (2015a), where smaller global and subfield hippocampal volumes significantly correlated with higher severity of childhood traumatization and dissociative symptoms. Dissociative amnesia has been found to correlate uniquely and significantly with reduced bilateral hippocampal subfield volumes (Chalavi et al., 2015a). Additionally, Dimitrova et al. (2023) have demonstrated that emotional neglect, a form of traumatization, exerts a particularly deleterious effect on hippocampal volume and is interlinked with dissociative amnesia (Figures 1–4). See Figure 1 illustrating structures of the brain impacted by traumatization and mentioned in the text, Figure 2 illustrating the cortico-basal-thalamo-cortical loops of the brain, Figure 3 illustrating the default mode network or DMN, and Figure 4 depicting the sensorimotor network, or SMN, and the central executive network, or CEN.

### The functional networks and the connectivity patterns of the traumatized brain

In their review of MRI studies of the brains of psychiatric patients, Lotfinia et al. (2020) noted that dissociative processing does not occur in a limited number of specific brain regions. Instead, it appeared associated with distinct neural signatures. Recent developments in brain research related to stress and trauma have focused on functional brain networks and how they potentially cooperate (Jin et al., 2023; Shaw et al., 2023). Traumatic experiences are hypothesized to have a widespread, cascading impact on the connectivity of the brain’s networks (Kearney and Lanius, 2022). Shaw et al. (2023) found that participants with PTSD of a dissociative subtype exhibited widespread functional hyperconnectivity among subcortical regions, sensorimotor-related networks, and other intrinsic connectivity networks compared to controls. In a separate study, Rabellino et al. (2015) identified altered functional connectivity within three large-scale intrinsic connectivity networks (ICNs) in individuals with PTSD while they processed threat-related stimuli. These networks include the Central Executive Network (CEN), also known as the Dorsal Frontoparietal Network (DFPN), as previously described by Uddin et al. (2019), the Salience Network (SN), and the Default Mode Network (DMN). The networks were found to be affected during both sub- and supraliminal processing of threat-related stimuli (Rabellino et al., 2015).

Lebois et al. (2021) intrinsic network connectivity analysis of functional MRI scans obtained from 65 women with histories of childhood abuse, current posttraumatic stress disorder (PTSD), and dissociation identified seven networks, thereby establishing a brain basis for trauma-related dissociation. Their connectivity estimates were derived from a novel machine-learning technique using individually defined homologous functional regions for each participant (Lebois et al., 2021). The identified networks included the Visual Network, the Sensory-Motor Network (SMN), the Dorsal Attention Network (DAN), the Ventral Attention Network (VAN), the Limbic System Network (Limbic or Salience Network, SN), the CEN, and the DMN. The severity of dissociative symptoms was found to correlate with the concurrent activation of the DMN and the CEN, according to Lebois et al. (2021). This pattern of hyperconnectivity has emerged as a new and significant predictor of severe dissociative symptoms in DID.

### Default mode network DMN

The default mode network (DMN) is involved in baseline mental activities, facilitating spontaneous introspection and recollecting the past and future. This capacity for introspective thinking and time travel is a hallmark of the DMN. When individuals ponder others, themselves, and/or moral or aesthetic subjects, such as the arts, their DMN is activated, and their brains shift their focus from the external world to daydreaming or restful wakefulness (Raichle, 2015; Raichle et al., 2001; Wen et al., 2022, 2024). Thus, the DMN plays a crucial role in self-awareness and understanding others (Menon, 2023; Raichle et al., 2001; Wen et al., 2022, 2024). The DMN consists of the medial PFC, the posterior cingulate cortex, the precuneus, the left and right parietal cortex, and the angular gyrus (Raichle, 2015). Research has consistently demonstrated a link between early trauma, disturbances in DMN regions, and psychopathology, according to Tian et al. (2022). Increased PTSD symptom severity has been shown to correlate with reduced connectivity between the anterior and dorsal parts of the DMN compared to those observed in healthy controls (Akiki et al., 2017; Kearney and Lanius, 2024). Altered functional connectivity within the DMN, modulated by the periaqueductal gray (PAG) located deep within the midbrain, appears to influence self-related processes. These findings have significant clinical implications. As demonstrated in the study by Terpou et al. (2020), participants with PTSD demonstrated clinical disturbance of DMN connectivity when processing trauma-related stimuli.

### The executive function networks of the brain, including the central executive network

The executive function networks of the brain, including the Central Executive Network (CEN), have been further delineated in the research by Witt et al. (2021). The executive function networks of the brain consist of four networks: the Dorsal Attention Network (DAN), the Left and Right Central Executive Network (CEN), and the Anterior Control Network (ACN). The CEN plays a pivotal role in executive function, cognitively demanding tasks, and decisions regarding goal-directed behavior (Uddin et al., 2019). The CEN enhances top-down attentional control, allowing for flexible division into the DAN for perceptual attention or another network, the DMN, involved in introspective processes. The CEN primarily comprises the dorsolateral PFC, the posterior parietal cortex, the frontal eye fields, and part of the dorsomedial prefrontal cortex (dmPFC) (Menon, 2011). Within the CEN, the severity of PTSD symptoms has been linked to the hyper-coupling of ventrolateral PFC, while the presence of depersonalization/derealization symptoms in PTSD of a dissociative subtype has been associated with overmodulation of limbic activity via ventromedial PFC hyperactivation (Murphy, 2023). Dimitrova et al. (2024) found that the CEN and DMN were activated simultaneously for autobiographical cues in relation to dissociative symptom severity in both CPTSD and DID clients, implying an internal reorientation of cognitive attention during self-referential processing with heightened cognitive control over self-relevant processing of memories. Individuals dealing with complex trauma and dissociation often exhibit resistance to recollecting memories, leading them to employ brain circuits that unconsciously divert attention from potentially harmful information. For a comprehensive review of related research, refer to the work of Dimitrova et al. (2024).

### Salience network

The SN plays a pivotal role in the perception of bodily sensations associated with reward and aversion, thereby modulating the switching between these states. The SN is a large-scale paralimbic–limbic network anchored in the anterior insula and dorsal anterior cingulate cortex. It has prominent subcortical nodes in affect and reward processing systems, such as the amygdala and the temporal poles (Menon and Uddin, 2010). The anterior insula and the anterior cingulate cortex separate and select the most relevant internal and external stimuli to guide behavior. The SN facilitates the transition between the default mode network (DMN) and the central executive network (CEN), and it is activated in response to various salient stimuli, including acute stress (Goulden et al., 2014). Stress detection reallocates resources to the SN, promoting fear detection and vigilance at the cost of the CEN (Hermans et al., 2014). Following the dissipation of stress, the focus reverses, thereby normalizing emotional reactivity and enhancing higher-order cognitive processes that are critical for long-term survival (Hermans et al., 2014). Individuals diagnosed with PTSD exhibited diminished thalamic functional connectivity, with the salience network (SN) and corticostriatal circuits being particularly affected (Peters et al., 2016). The cortico-striatal circuits (connections from the cortex to the midbrain and up to the cortex again) of the SN are important for appropriate goal-directed behaviors and motivation and may contribute to visual fear processing in PTSD through pathways from superior colliculi in the midbrain to pulvinar-mediodorsal thalamus-amygdala pathways (Yoshii, 2021). Disturbances in the cortico-thalamo-cortical circuits can result in self-perpetuating cycles of rumination, obsessive thoughts, and a hollow sense of bodily self (Corrigan and Christie-Sands, 2020).

### Connectivity patterns related to disturbances in experiencing a coherent and embodied self

The following section will explore connectivity patterns related to disturbances in experiencing a coherent and embodied self. According to Purcell et al. (2024), conditions diagnosed as PTSD, Complex PTSD (CPTSD), PTSD of a dissociative subtype, and severe dissociative disorder DID involve dysregulation of the brain’s functional networks. These conditions are also associated with a decreased capacity for self-awareness and for being present in the here and now. In healthy subjects, the SN functions as a switch between the DMN and the CEN; however, in severe dissociation, the DMN and the CEN appear to be simultaneously activated (Lebois et al., 2021). Furthermore, due to facilitated threat detection circuitry, complexly traumatized persons may perceive innocuous stimuli as threatening (Harricharan et al., 2021).

Individuals struggling with the after-effects of protracted and profound trauma are susceptible to depersonalization and the re-emergence of distressing memories, as described by Kearney and Lanius (2024). This may be attributed to the disconnection between the anterior and posterior components of the DMN, which hinders the integration of information into a coherent autobiographical narrative. This dysfunction is believed to stem from poor cooperation between the anterior and posterior DMN, and the posterior DMN being hyper-coupled with the sensorimotor network (SMN) (Kearney et al., 2023b). Furthermore, the ability to withstand traumatic experiences disrupts the normal functioning of the thalamus, likely due to sensory overload originating from deeper brain regions (Walker et al., 2018). Consequently, the brain’s capacity to process traumatic experiences appears to be impaired, resulting in an unprocessed and ongoing experience of danger. This phenomenon often leads to unconscious coping mechanisms in traumatized individuals, such as avoidance of sensory overload through dissociation or numbing. While most of the research on DID has been conducted on patients with comorbid post-traumatic stress disorder (PTSD), individuals with DID exhibit more severe depersonalization, involuntariness, and instability between self-states compared to those with PTSD alone (Lotfinia et al., 2020; Loewenstein et al., 2024; Purcell et al., 2024). This discrepancy may be attributable to the specific impact of dissociation on network connectivity. How such changes in the developing brain may be reversible through therapy is not yet fully understood and will be the subject of further research.

Structural and functional alterations in the cerebellum have been linked to exposure to early adverse experiences, trauma-related psychiatric symptoms, and altered cerebellar connectivity to the brain’s macroscale networks, including the DMN, SN, and CEN (Blithikioti et al., 2022). The flocculus, a deep cerebellar nucleus, has been identified as a key player in motor control and gaze stabilization (Kheradmand and Zee, 2011). It is believed to play a critical role in the neurocircuitry that facilitates the experience of a coherent, embodied self. A review of the literature reveals that the flocculus appears to be compromised in cases of PTSD, particularly in the context of PTSD of a dissociative subtype. Compared to individuals without a history of trauma, those with PTSD and PTSD of a dissociative subtype exhibited reduced resting-state functional connectivity between the left flocculus and the cortical regions involved in bodily self-consciousness (Rabellino et al., 2023).

The hyperconnectivity of the DMN and the CEN may underlie the failure to adaptively integrate aspects of identity and consciousness (Menon, 2021). Although the individual may face significant challenges, excess corticolimbic inhibition may, according to Purcell et al. (2024), help the PTSD of a dissociative subtype or DID person not feel the body. Consequently, when the active defenses transition to a passive state, the individual can undergo a state of mental disengagement and/or avoidance of interoception. This unconscious strategy may temporarily alleviate pain and suffering, yet it can result in an elevated threat response. A hypersensitization of the thalamic-cortical-amygdala pathway, or a direct midbrain-thalamo-amygdala pathway, has the potential to perpetuate this cycle (Kearney et al., 2023b; Kearney and Lanius, 2024), leading to heightened reactivity to both prosaic and potentially triggering stimuli. The hyperconnectivity between the PAG in the midbrain and the posterior part of the DMN, precuneus, might, in conjunction with diminished differentiation of the SMN and the posterior DMN (Kearney et al., 2023b), elucidate why unintegrated experiential residues frequently inundate the traumatized individuals and further destabilize their perceptual systems. This phenomenon provides a plausible neural basis for the re-experiencing of flashbacks or decontextualized flashbacks, often accompanied by somatosensory components that emerge spontaneously and persistently in individuals with CPTSD and/or severe dissociation (Kearney and Lanius, 2024).

Lyu et al. (2023) utilized single-pulse electrical stimulations and neuroimaging and found that specific sites within the anterior precuneus induced dissociative alterations, engendering experiences such as floating, elevation, and self-dissociation, thereby partially disrupting the sense of self. The anterior precuneus plays a pivotal role in the experience of “I.” The DMN contributes to the formation of “me” through integrating and broadcasting memory, language, and semantic representations to create a coherent “internal narrative” reflecting individual experiences (Menon, 2023). Such a narrative is central to the construction of a sense of self (Menon, 2023). These regions, in conjunction, are instrumental in shaping the “self” perspectives necessary for the development of a theory of mind (Di Plinio et al., 2020). In severe posttraumatic conditions and dissociation resulting from trauma, these structures are often compromised.

### The defense cascade in threat appraisal

During traumatic experiences, deeper brain parts become involved and dysregulated, including the PAG and extended brainstem systems (Corrigan and Christie-Sands, 2020). This dysregulation may result in changes in the brain’s functional networks, including the cortices, the limbic system, and the cerebellum, according to Terpou et al. (2019a,b), Terpou et al. (2020), Lebois et al. (2021, 2022), Lotfinia et al. (2020), and Shaw et al. (2023).

Typically, the cerebral cortex and limbic regions, such as the amygdala and the PAG in the midbrain, maintain a balanced relationship and are capable of flexibly influencing the activity of the other. Brain regions like the PAG are involved in both positive and negative valence and emotional processes (as is the amygdala). These structures play a role in alerting the individual’s attention to relevant information. The PAG has been shown to play a role in non-conscious emotion perception and reflexive behavior aimed at helping to escape from threatening situations (Terpou et al., 2019a,b). In situations where the source of dysregulation is near, such as in cases of attachment ruptures and/or interpersonal violence (Mobbs et al., 2007), the cascading influences from the PAG on arousal and affect regulation and higher-order capacities are substantial (Terpou et al., 2019b). The defense cascade (Kozlowska et al., 2015) delineates the startle response initiated in threatening scenarios, facilitating the individual’s orientation toward the threat (Terpou et al., 2019b). When the threat is perceived as hazardous, the individual shifts into active defensive mechanisms, such as flight or fight. Conversely, if the threat is perceived as inescapable and evaluated as life-threatening, the person shifts into a state of tonic immobility, where a shift from our inborn active defenses turns into passive defenses. These responses, ranging from the startle response to the total submission, are initiated by the different columns of the PAG located in the midbrain. The involvement of the PAG in implementing rapid shifts in attentional vigilance, autonomic state, and defensive behaviors is related not only to activation but also to its functional connectivity with cortical regions such as the insula, ACC, and medial prefrontal cortex.

Activation of the dorsal and lateral columns of the PAG has been associated with active defense behaviors, such as fight and flight. In contrast, the ventrolateral columns have been linked with passive defense behaviors (Terpou et al., 2019a,b). The activation patterns of PAG can culminate in a state of numbing and collapse, wherein the release of endogenous opioids mediates reduced arousal, dissociation, and analgesia (Lanius et al., 2020; Terpou et al., 2019b). Consequently, therapeutic interventions for traumatized individuals could aim to target the PAG in the midbrain and associated structures, irrespective of whether dysregulations of these regions are attributable to traumatic experiences present or past.

## Addressing depersonalization in trauma therapies

Dissociative conditions are frequently typified by a vague sense of ongoing self and depersonalization (Ferroni et al., 2024; Simeon and Abugel, 2006; Murphy, 2023). Depersonalization is characterized by a feeling of unreality or alienation around the self and the environment. In DID, depersonalization stems from experiences of helplessness, hypoarousal, and lowered levels of consciousness (Loewenstein et al., 2024), variously termed “total submission,” “playing dead,” or “collapse” (Terpou et al., 2019a,b). These phenomena coincide with the endpoint of the passive defenses of the trauma cascade.

The prevalence of depersonalization is notably high among individuals with a history of interpersonal abuse, with estimates ranging from 25 to 53.8% (Yang et al., 2023). The impact of trauma and neglect on the developing brain is substantial, with lifelong implications (Teicher et al., 2022). Subsequent maltreatment and traumatization alter trajectories of brain development (Bremner, 2006) and impact sensory systems, network architecture, and circuits involved in threat detection, emotional regulation, and reward anticipation (Schore, 2009; Teicher et al., 2016). Therapy should address these dysregulations while exploring how depersonalization develops or resolves following treatment and identifying effective methods for highly traumatized and dissociative clients. (Jin et al., 2023) and shift the negatively valenced PAG toward more positive emotional states (Damasio, 1998; Panksepp, 1998; Terpou et al., 2019a,b; Terpou et al., 2020).

### Therapy for complexly traumatized clients and those with dissociative disorders

The most studied treatments for PTSD are various forms of CBT and EMDR (Khan et al., 2018). However, given the paucity of evidence supporting the efficacy of alternative treatments, the recommendation is limited to evidence-based interventions. Nonetheless, there is a call from several researchers for the development of additional therapeutic modalities, particularly for those dealing with complex trauma and dissociation (International Society for the Study of Trauma and Dissociation, 2011; Holbæk et al., 2024; Nijenhuis, 2017; van der Hart et al., 2017).

Grossman et al. (2017) found that of the nearly 100 distinct evidence-based or promising practices identified, none have been designed to target the effects of childhood emotional abuse and neglect in adults or child survivors. This finding may be a contributing factor to the fact that more than 30% of PTSD clients do not benefit from evidence-based treatments (Schouten et al., 2019). Imel et al. (2013) found that meta-analyses revealed that approximately 40% of PTSD patients retained their diagnosis following treatment with trauma-focused cognitive behavioral therapy (TF-CBT), with high rates of attrition observed in exposure therapies exceeding 30%. However, a recent study revealed that over 80% of the clients no longer met the PTSD and CPTSD diagnostic criteria after undergoing intensive trauma-focused treatment, with no recorded dropouts (Bongaerts et al., 2022). De Jongh and Hafkemeijer (2024) presented a case report with a CPTSD client who also had BPD. No stabilization was undertaken before a short-term EMDR 2.0 (Matthijssen et al., 2021) was successfully used. EMDR 2.0 is a version of EMDR that focuses on more ongoing sensory activation.

Nevertheless, many researchers believe that those who have PTSD, and especially more complex forms of traumatization, benefit from a phase-oriented treatment approach, in which the client’s safety and coping skills are established before working directly with traumatic memories (Herman, 1992). The first phase, Phase 1, symptom reduction and stabilization, aims toward overcoming phobias of mental contents, dissociative parts, and attachment and attachment loss with the therapist (Steele et al., 2005). Several grounding techniques are used during this phase (Brand et al., 2022; Boon et al., 2011). This approach is not only recommended but also supported by empirical evidence (Willis et al., 2023). Its application to individuals suffering from CPTSD is equally substantiated, given the adaptation of existing trauma-focused treatments to address the impairing DSO symptom clusters (Jowett et al., 2021).

The seemingly contradictory findings may be attributable to varying unconscious strategies of information processing among different categories of traumatized individuals, potentially resulting in distinct functional network connectivity patterns. Del Río-Casanova et al. (2016) proposed a hypothesis concerning a gradient of over- and under-regulation, with under-regulated clients (BPD, PTSD, and CPTSD) occupying one extreme of the continuum and clients with somatoform and psychoform dissociative disorders (predominantly exhibiting negative symptoms and emotional shutdown) occupying the other extreme. The oscillatory nature of over- and under-regulation has been demonstrated in DID cases, as Lebois et al. (2022) reported. Schlumpf et al. (2021, 2022) observed that phase-oriented treatment for clients with CPTSD and/or dissociative disorders enhanced the resting-state network connectivity of the default mode network (DMN) and the connectivity between brain regions activated during autobiographical recall. This enhanced their ability to cope with emotional challenges. Following treatment, the connectivity of traumatized clients’ brains in the networks associated with cognitive control and memory was normalized relative to healthy controls (Schlumpf et al., 2019).

### Therapy for clients with DID

The therapeutic approach for individuals diagnosed with DID is a subject of ongoing research. Studies postulating the efficacy of psychotherapy in inducing structural and functional changes in the brains of individuals with severe psychopathology are undertaken (Malhotra and Sahoo, 2017). Schlumpf et al. (2021, 2022) found that phase-oriented treatment could enhance functional connectivity between regions activated during autobiographical recall in patients with complex trauma and dissociative disorders. However, research on the effects of psychotherapy in people with DID remains limited. For those more severely traumatized, such as DID patients, the focus of exposure therapy can be unclear and potentially contradictory (Loewenstein et al., 2024). Theoretically, when treating DID clients, a brain-informed, consciously resource-activating therapeutic approach is recommended to buffer the overload of negative effects (Gerge, 2018a). The necessity of psychotherapy interventions to mitigate experiential avoidance and disrupted information processing in DID sufferers is well-supported by the extant literature (Lanius, 2015; Purcell et al., 2024). The importance of an extended stabilization phase, with a focus on skills to regulate emotions and arousal, is also highlighted (Boon et al., 2011), including the development of skills to regulate dissociative states (Brand et al., 2022; Loewenstein and Brand, 2023). Therapeutic interventions should address dissociation and its potential impact on the central nervous system. Dissociative processes are theorized to impact various brain levels, including the cerebellum, midbrain, and cortex, as well as the brain’s functional networks, as suggested by Dimitrova et al. (2024) and Purcell et al. (2024). Addressing these dysregulations through therapeutic interventions is crucial for effective treatment (Schlumpf et al., 2022).

The recommended treatment modalities for DID, informed by insights from neurobiology (Purcell et al., 2024), advocate a phase-oriented treatment approach (Herman, 1992; Brand et al., 2022), clinical hypnosis (in accordance with the guidelines of the International Society for the Study of Trauma and Dissociation, 2011; Kluft, 2012; Loewenstein and Brand, 2023), and potentially DBR (Corrigan and Christie-Sands, 2020). The treatment supposedly needs to address two aspects: (i) dissociative self-states (Kluft, 2012; Loewenstein and Brand, 2023; Loewenstein et al., 2024) and (ii) the trauma-induced changes in the brain’s intrinsic neuronal networks (Jiang et al., 2017). The extreme avoidance of traumatic material in dissociative disorders is probably best dealt with within a trusting therapeutic relationship.

DID has been linked to increased connectivity between the CEN and DMN (Lebois et al., 2021). Clinical hypnosis has been demonstrated to reduce hyperconnectivity between these networks in nonpsychiatric control participants (Jiang et al., 2017), and clinical hypnosis has been recognized as a viable treatment modality for individuals with high hypnotizability, including those with DID (Dell, 2009; Kluft, 2012; Loewenstein and Brand, 2023; Loewenstein et al., 2024).

#### Art therapy focuses on resourcing

In the context of working with clients who exhibit high levels of dissociation, the utilization of resource-oriented metaphors has been identified as a potential strategy to facilitate stabilization in their therapeutic processing (Frederick and McNeal, 1998). The employment of inner strength imagery, in conjunction with positive or soothing imagery and relationally held suggestions for altered attentional focus, has been proposed to enhance outcomes (Gerge, 2018a). Such states can be induced through art-based protocols that emphasize agency and change (Hass-Cohen et al., 2014), aiming for memory reconsolidation (Hass-Cohen and Clay, 2025a) and resourcing. The experience of heightened safety has been shown to enhance clients’ well-being and introduce more positive states of mind (Ruysschaert, 2014). When accessing and regulating implicit processing and trauma-bound intrusions (Kearney and Lanius, 2024), creative arts therapy interventions, such as AT, can facilitate coregulation in the implicit domain and resource activation. The profound experience of loneliness of the attachment-wounded client might be addressed by engaging in art-making with the therapist or by enhancing relational holding during the art-making process. However, it is important to note that the implementation of these techniques in the context of highly dissociative clients necessitates a gradual and incremental approach, akin to the metaphorical “one drop at a time” (Kluft, 1990a,b, 2012), as facilitated by the therapist’s adept use of relational metaphors and plain hypnotic techniques. These techniques can be employed in Active Alert Hypnosis (AAH) (Bányai, 2018) and induced through the arts, as focused attention often induces altered states of consciousness in highly dissociative clients, whether highly hypnotizable or not (Kasos et al., 2018). AAH, combined with the use of positive imagery, imagined or depicted, can facilitate increased executive attention and emotional control in the present and future, thereby strengthening” happy pathways” in the brain (Ruysschaert, 2014). Consequently, interventions aimed at reregulation and fostering hope can (re)establish a sense of safety and fostering hope (Gerge, 2018a). They can potentially reinstate a phenomenological experience (Gendlin, 1978) of safety, which can mitigate heightened arousal, as indicated by criteria B and E of the PTSD diagnosis. The restoration of self-soothing capacity, as defined by Krystal and Krystal (1988), can lead to a shift in both negative inner schemas and trauma-bound cognitions, thereby mitigating criterion D of the PTSD diagnosis. It is important to note that these processes, over time, often necessitate engagement with varying states of being in a highly dissociative client.

#### Deep brain reorienting

Preliminary recommendations have been made for implementing Deep Brain Reorienting (DBR) (Kearney and Lanius, 2024; Purcell et al., 2024). To date, no studies have been conducted on DBR in the treatment of DID. However, the results from an RCT (Kearney et al., 2023a) on PTSD and CPTSD are encouraging, and the theoretical model has been deemed viable. DBR is a trauma psychotherapy that aims to facilitate the processing of traumatic experiences. DBR facilitates the tracking and re-focusing of the original sequence of physiological responses that occurred when structures in the deep brain were alerted to a threat or an attachment disruption. These structures comprise the superior colliculi, the PAG in the midbrain, and the locus coeruleus in the brainstem. It is assumed that DBR has the potential to reverse the impact of traumatization on appraisal processes in the lower-level brain regions. This approach enables individuals to regain a more balanced regulation of their internal environment (Corrigan and Christie-Sands, 2020; Corrigan et al., 2025; Kearney et al., 2023a). Guided bodily awareness as a vehicle for integration and transformation is not a novel concept (Gendlin, 1978, 1992; Levine, 2010; Ogden et al., 2006). However, DBR differs and goes beyond invoking mindfulness of bodily reactions (Kearney et al., 2023a). The precision with which DBR is applied in relation to the brain’s appraisal processes can ameliorate feelings of being overwhelmed and states that otherwise would potentially result in clinical deterioration. DBR assists clients in maintaining presentness and averting unconsciously evasive responses to their experiences. Experiences that the clients previously unconsciously considered overwhelming (Corrigan and Christie-Sands, 2020).

## Mechanisms of change in art therapy for traumatized clients

Traumatization, defined as an assault on the senses and a breakdown of interpersonal safety, highlights ATs’ transforming capacity through the formation or reformulation of sensory-motor feedback loops that foster a sense of agency, power, and a positive self-other relationship (Lanius et al., 2011). AT’s sensory-based antidote, in principle, has the potential to modify trauma-bound flashbacks and intrusions into autobiographical memory (Kearney and Lanius, 2024; Hass-Cohen and Clay, 2025a). Potentially, a process occurs when memories are triggered, reprocessed, reconsidered, and reintegrated into long-term memory (Elsey and Kindt, 2017; Elsey et al., 2018). Then arts-based processing help in the present moment, fostering agency, coherence, and a sense of belonging to both themselves and the world (Damasio, 2003, 2012). AT has been shown to facilitate an augmented relational capacity, particularly regarding the self (King et al., 2019), and may potentially modify the neurophysiological alterations associated with traumatization (Payano Sosa et al., 2023).

Creating art can be an outlet for clients’ affects and states that are otherwise difficult to reach (Estrada Gonzalez et al., 2024; van der Kolk, 1994; van der Kolk and Fisler, 1995). Consequently, subjects who have experienced a paucity of joy, pleasure, and triumph (mastery of challenging interpersonal situations) in the context of ongoing trauma can once more experience these emotions (Williams et al., 2018; Payano Sosa et al., 2023). In RAT, the therapeutic process involves engagement with the attachment system (Gerlitz et al., 2020). When these experiences are embodied and relationally held, it can be understood that traumatic experiences have ended. Then the phenomenon of trauma-induced awake trance states (Gerge, 2009) is observed to undergo a reduction in intensity when exposed to ongoing sensory input and can culminate in the realization of completed acts.

The “trauma cascade” (Kozlowska et al., 2015; Terpou et al., 2019b) has been shown to induce heightened arousal, submission, collapse, and negatively loaded effects. However, recent studies have indicated that these effects can be mitigated by implementing resourcing imagery via AT (Gerge, 2017, 2018b). AT offers a tangible presence that can be instrumental in the treatment of severe post-traumatic and dissociative conditions, as the approach has been shown to facilitate emotion processing (Czamanski-Cohen and Weihs, 2023; Haeyen et al., 2018; Malhotra et al., 2024).

The integration of aesthetic experiences within therapeutic interventions has been demonstrated to modulate traumatic memories, leading to a reduction in pathological arousal (King and Chatterjee, 2024). Furthermore, AT offers a rich source of contextual information, a crucial aspect of arts-based therapies (Vaisvaser et al., 2024). Updating fear-based memories with non-fearful information through arts-based therapy can result in a permanent reduction in automatic avoidance responses and enduring changes in distressing memories (Rudstam, 2023). This process can involve activities such as drawing, where the integration of hand, eye, and intention is promoted. Other embodied and relationally held expressive arts therapy interventions (Malchiodi, 2020) can be utilized.

The artistic expression conveys a piece of condensed reality, where we can become visible to ourselves and others (Vaisvaser, 2021, 2024). Thus, AT might enhance neurophenomenological learning about the self (Vaisvaser, 2021, 2024), also when the learning is compromised by the impact of traumatization. This learning is purportedly founded on relationally held neuro-aesthetic principles (Oliva et al., 2023; Vaisvaser et al., 2024; van Leeuwen et al., 2022) and can result in an augmented integrative capacity. This heightened capacity may, in turn, result in a reduction of PTSD symptoms through a tolerable form of exposure that facilitates adaptive sensory perception and stress regulation (Malhotra et al., 2024; Payano Sosa et al., 2023). AT has been referred to as “neuropsychology in action” (King et al., 2019), yet the extant research substantiating this claim is insufficient.

Despite the ongoing research on the effectiveness of trauma-focused art therapy for psychological trauma (Heijman et al., 2024; Payano Sosa et al., 2023), there is a paucity of quantitative studies in AT. Moreover, the theoretical foundation for the protocols is considered weak (Baker et al., 2017; Oliva et al., 2023; Schouten et al., 2015). The evidence level is considered low, as evidenced by the paucity of RCTs conducted on AT, with only one RCT for combat-related PTSD (Campbell et al., 2016). Additionally, methodological shortcomings have been identified in the design of published papers (Schnitzer et al., 2021). However, a recent RCT (Rudstam et al., 2022) on trauma-focused group music and imagery, with artmaking incorporated into the treatment protocol for women with PTSD/complex PTSD, found a significant effect for the intervention concerning reduced PTSD and CPTSD symptoms. Moreover, in an RCT, AT was found to be an effective treatment for personality-disordered patients, a group with a high amount of childhood trauma. AT reduced pathology and maladaptive modes and helped the patients develop adaptive and more positive modes, which might indicate better mental health and self-regulation (Haeyen et al., 2018).

While more robust research is necessary, moderately reduced trauma-related symptoms were reported in individuals with PTSD who received AT (Baker et al., 2017). A combination of AT and EMDR is being researched to find a more accessible treatment form for traumatized persons (Tripp, 2023). However, the correspondence of the arts in therapy with the emerging understanding of the impact of psychological trauma and dissociation on the brain’s anatomy, functional networks, and connectivity patterns in processes of healing is yet to be researched.

### Art therapy in the treatment of dissociative identity disorder

The present study aims to explore the potential of art therapy in the treatment of DID. As previously mentioned, implicitly stored trauma memories appear to be contextualized and integrated through the arts-making process (Hass-Cohen and Clyde Findlay, 2015; Hass-Cohen et al., 2018; Hass-Cohen et al., 2018; Hass-Cohen and Findlay, 2019; Hass-Cohen and Clay, 2025a; Perryman et al., 2019; Vaisvaser et al., 2024). However, for clients with DID, only a limited number of case studies have been conducted on AT (Gerge, 2018c; Loewenstein et al., 2024; Somer and Somer, 1997).

Accurate diagnosis is crucial when using arts-based interventions with highly dissociative clients (Loewenstein et al., 2024; Kluft, 2005; Mitra and Jain, 2024). When working with a client with DID who has not been diagnosed with DID, the manifestation of negative emotions, numbing, or the onset of speechless terror and pain can occur in response to interventions that have been demonstrated to be effective for other clients. Growing research on brain network connectivity suggests that several networks are influenced by engaging in or experiencing art (Di Plinio et al., 2020; Hutton, 2014; Vaisvaser et al., 2024). Art experiences conveyed a sense of being “touched from within” that other external stimuli did not normally activate (Vessel et al., 2013). Then, therapists must recognize that the act of requesting that a highly dysregulated DID client create an image of being vulnerable or a drawing that captures moments of fear, loneliness, or powerlessness can potentially activate a condition of being “touched from within” in a too overwhelming manner. Such activation can lead to self-discovery previously avoided, potentially for years or even decades. However, when such inquiries are met with sufficient relational support, carefully tailored to the individual’s needs, and executed with gentleness, they have the potential to facilitate changes in states of being. These changes may, in theory, be attributed to therapy-induced heightened neuroplasticity.

Enhancing dissociative clients’ capacities to self-reflect and be more present in the here and now is considered a prerequisite for trauma work with DID clients (Loewenstein, 2023). Theoretically, AT-based interventions can stabilize these clients’ information processing. The externalization-concretization process, involving the use of colors, shapes, and compositions, has been proposed to assist complexly traumatized and dissociative clients who exhibit impaired physiological regulation (Vaisvaser et al., 2024). This approach is further supported by the instruction to clients to create visual representations of safe spaces, protective figures, and metaphorical shelters (Gerge, 2018c; International Society for the Study of Trauma and Dissociation, 2011; Purcell et al., 2024).

However, the art therapist needs to acknowledge that the visual depictions produced by these clients can be profoundly evocative, potentially triggering dissociative responses in other states of being of the client. In the context of DID treatment, the art therapist must be cognizant of the potential impact of these visual materials on the therapeutic relationship and the client’s integration process of being touched from within. The therapeutic relationship can deepen through the therapist’s capacity to protect the client from being overwhelmed by previously unbearable information. For example, states of being—parts that might otherwise be overwhelmed when traumatic experiences or states are explicitly drawn and made visible—can be asked to look very briefly at the picture, or the therapist can gently tell the client what they see. The metaphor of back-and-forth-looking binoculars that reduce the image can be used, or the therapist may have agreed with the patient that parts of the personality, for example, very young or vulnerable parts, do not need to be active during a specific session. A relationally attuned therapist who uses soothing metaphors and interventions aimed at heightened agency is needed. Then, moving from intrusions and depersonalization to agency in the here and now, in a safe enough manner, can take place.

In the treatment of DID clients, their altered states of consciousness (Frewen and Lanius, 2015) need to be addressed. These are also labeled self-states and self-state systems (Loewenstein, 2023; Loewenstein and Brand, 2023), states of being (Putnam, 2016), or trauma-bound ego states (Watkins, 1978; Watkins and Watkins, 1997). Self-states are embodied, dynamic constructs grounded in perception, action, and introspection based on experience (Putnam, 2016; Lebois et al., 2023). These aspects of the self can be expressed in several ways. For example, they can be expressed through embodiment (as in somatoform dissociation; Nijenhuis et al., 1997, 1999) or through acting out of the dynamics of disorganized or otherwise insecure attachment patterns (the phobia of attachment and the phobia of attachment loss (Liotti, 2013) and/or malevolent appearances (Watkins and Watkins, 1988) when identified with the aggressor (Howell, 2014). Additionally, the theory of structural dissociation (Nijenhuis and van der Hart, 2011; Steele et al., 2005) provides a perspective that incorporates the concept of part-selves.

As Loewenstein (2023) and Loewenstein and Brand (2023) posited, trauma-generated self-states can be accessed, thereby initiating a shift in their worldviews through implicit processing, most often facilitated by clinical hypnosis. However, it is noteworthy that visual mediums such as pictures and sculptures have been observed to serve as conduits into the clients’ frequently frightened inner realms. This observation suggests the potential for relational art-making to attain similar domains. Dimitrova et al. (2024) have proposed heightened cognitive control and inter-identity avoidance of trauma-related knowledge in DID, as the CEN of their brains appears, potentially, to be used to *not* know about the self. Consequently, depersonalization/derealization in DID can be conceptualized as an exaggerated emotional or arousal response leading to an excessive corticolimbic inhibition, where the regulatory mechanisms that control emotional and arousal responses are excessively suppressed in the brain (Purcell et al., 2024).

In line with the recommendations of Dimitrova et al. (2024), treatment for individuals with DID should prioritize emotion regulation, grounding, and safe stabilization, aligning with the approaches proposed by Brand et al. (2022) and Herman (1992). The implementation of grounding techniques can be facilitated through the arts in therapy. For example, colors that the client defines as soothing, often green, blue, or yellow in light shades (Gerge, 2018d), can help the client not destabilize. In addition, the use and depiction of whole and healing metaphors, such as” the well,” the (healed) tree,” or “you beyond the wounds” (Gerge, 2018a,b), can be valuable. Further, the arts can be used for energizing when helping dissociative clients not to collapse, as in relationally held scribbling or drumming (Gerge et al., 2019). Subsequently, in collaboration with the client, the therapeutic process can address and modify the psychological barriers that hinder the cognitive avoidance of traumatic material, whether derived from artistic or other sources. The reforming and re-establishing of implicit knowing can be enhanced not only through the careful application of the therapeutic relationship and arts-based interventions in therapy but also through methods explicitly reaching and re-regulating deep levels of the brain (Corrigan and Christie-Sands, 2020; Lebois et al., 2022; Purcell et al., 2024).

### Being touched from within

**Figure 5:**
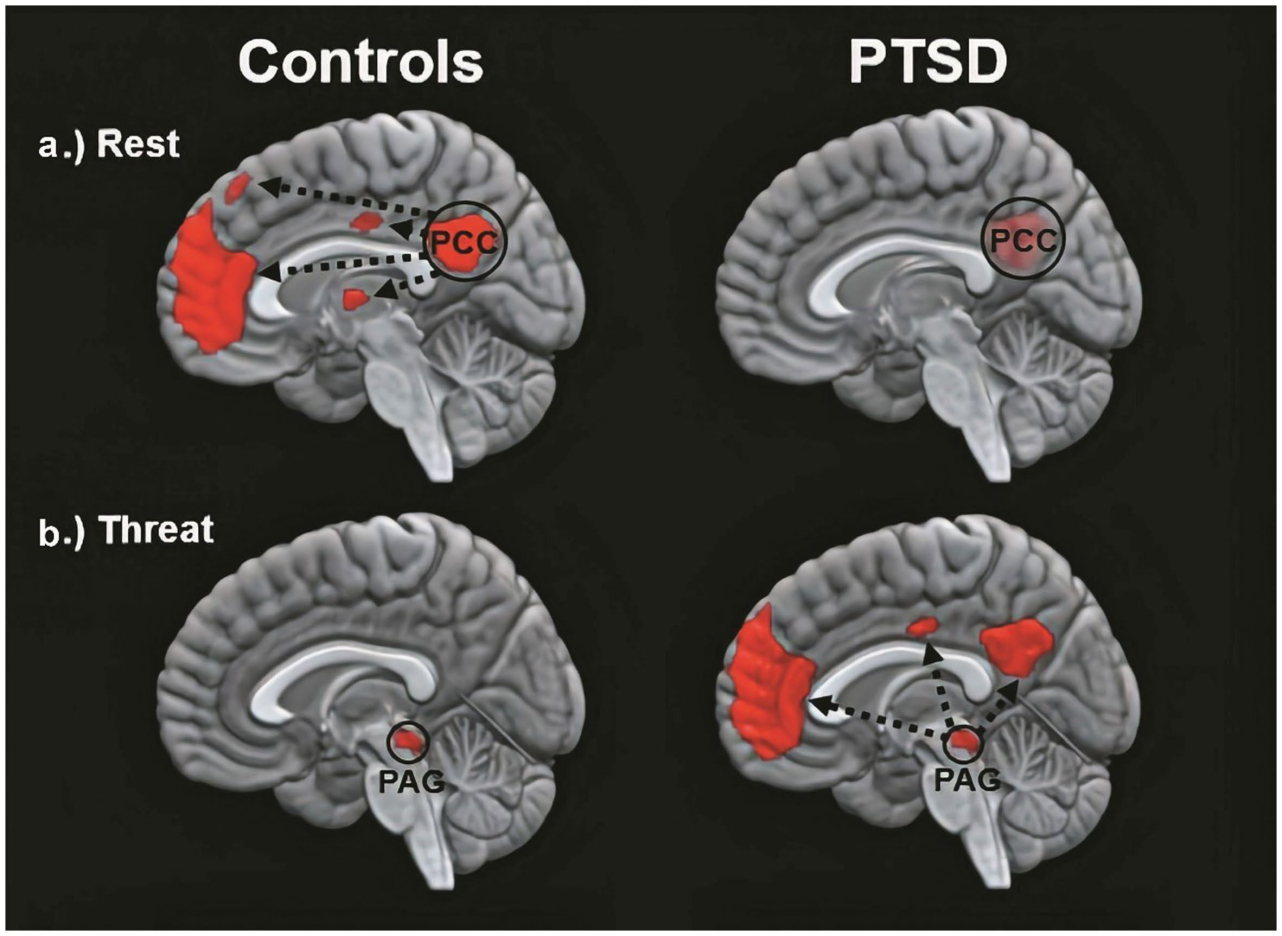
The functional connectivity of the DMN in healthy controls (left) and in participants with PTSD (right) varies according to different conditions. The top and bottom images depict within-group patterns in functional connectivity during rest and during trauma-related stimulus processing, respectively. This figure has been adapted from Bluhm et al. (2009) and Terpou et al. (2019a, 2019b). As demonstrated in the figure, the traumatized brain exhibits impaired ongoing self-referential processes during rest and transitions into a trauma-induced awake trance state under conditions of perceived stress (Lanius et al., 2020). This figure was kindly provided by Professor Ruth Lanius, MD, PhD, FRCPC.

In a study by Bluhm et al. (2009), changes in the DMN and self-referential processing were observed in patients with chronic PTSD related to early-life trauma when compared to healthy controls. One hypothesis is that AT experiences, when adapted with care, have the potential to regulate and soothe traumatized individuals by reaching, activating, and regulating the DMN (Vaisvaser et al., 2024). In severe post-traumatic conditions, the contact between the anterior and posterior nodes of the DMN is often impeded by hypoconnectivity (Kearney et al., 2023b). As illustrated in Figure 5 by Lanius et al. (2020), the functional connectivity of the DMN in healthy controls differs significantly from that in participants with PTSD under rest and threat conditions.

When a non-traumatized person experiences the arts, the anterior and posterior nodes of the DMN are said to be co-activated (Osuch et al., 2009; Bolwerk et al., 2014). This appears to be related to psychological resilience, e.g., increased stress resistance (Bolwerk et al., 2014; Vaisvaser et al., 2024). King and Chatterjee (2024) state that the CEN, DMN, and SN are the three most relevant functional networks in aesthetics and creativity. Consequently, AT interventions can be studied through neuroscience, providing an impression of a person’s state of mind (King and Chatterjee, 2024). Payano Sosa et al. (2023) found the thematic content of artworks conducted by military service members with PTSD correlating with their self-report scales, symptom checklists, and fMRI scans. The scans analyzed the resting state connectivity of DMN and related brain region connectivity. The PTSD patients who depicted psychological closure and/or healing in art therapy demonstrated alterations in resting state functional connectivity across regions associated with attention, memory, pain processing, and language (Payano Sosa et al., 2023).

However, it is not just a matter of “activating the DMN” or unreflectively activating increased connectivity between the DMN, sensorimotor cortices, and other functional networks (Belfi et al., 2019). Such patterns of activation, although potentially beneficial for non-traumatized populations, may be the very reason why DID clients fear their own images, although this remains to be explored. The DMN appears to track the internal state of the person engaged in an aesthetic experience (Belfi et al., 2019). When a hitherto dissociated experience becomes too quickly and overwhelmingly “real,” the tendency to disengage may end any possible processing.

Such a tendency may be rooted in early experiences of being overwhelmed and not being helped to regulate. We thus need to offer our clients finely tuned help so they can be present with their sensations again. Then, their brain functioning can change towards less depersonalization, more appropriate threat perception, and increased resourcefulness. However, the extent to which the art-making process contributes to activating the orienting response, synchronized relational holding, or changes in interoception and inner imagery has not been explored. Nor has it been explored whether any of these are potentially beneficial changes enhanced by AT, and if AT offers reduced anxiety activation by activating the retinotectal pathway (superior colliculus-pulvinar-amygdala connection) (Yoshii, 2021). The pulvinar is thought to be a “visual” thalamic nucleus that contributes to visual attention, processing of emotional stimuli, eye-hand coordination, and manual grasping (Mitchell et al., 2023).

## Case vignette

“Many people fear finding themselves alone, and so they don't find themselves at all.” May (1982)

Regarding this case, the client has given informed consent to the publication of material describing some change processes during the last 2 years of therapy where relational AT and DBR have been used together in sessions. The therapy is ongoing, and the client kindly agreed to be interviewed by the second author and to share what she felt was working for her in the psychotherapy. After the vignette, the results of the Participatory Action Research, PAR, semi-structured interview (Purcell et al., 2024) are summarized.

Clara (pseudonym) is a middle-aged woman with a family, children, and work. She started therapy 15 years ago on the advice of her psychiatrist. Clara was clinically depressed and on medication. She suffered from a severe flight phobia and had previously been on sick leave due to burnout. Clara then attended two CBT treatments, though “the therapists never asked the right questions.”

Initially, she scored high on dissociation [12 on the SDQ5 (Nijenhuis et al., 1997), 17.1% on the DES and 10% on the DES-T (Bernstein and Putnam, 1986)], anxiety, and depression (19 on the HSC-25 A and 39 on the HSC-25 B; Derogatis et al., 1974). These figures did not reflect her severe dissociation, although SDQ5 was above the cut-off for a dissociative disorder. On the HSC-25 B, she scored 39, which indicates depression despite ongoing medication, as Clara was and is medicated. It was not until years later in therapy that the depth and complexity of her attachment wounds and debilitating dissociative symptoms were recognized. Clara’s outwardly functional life and her reticence to discuss her challenges likely contributed to this. She was then diagnosed with DID, according to the SCID-D (Steinberg, 1993).

Previously, we had worked through her CPTSD A criteria and related symptoms stemming from an abusive teenage relationship in which she suffered severe interpersonal violence. However, most of our time has been spent on relational psychodynamic work aimed at reducing her dissociative symptoms, symptoms that stem from severe childhood neglect and attachment wounds. Her dissociative vulnerability may have been the underlying factor contributing to the marked physical and psychological strain experienced during prior EMDR sessions despite their documented benefits. Clara then became very dysregulated again in sessions and between sessions, and the phase 1 work, stabilization (Herman, 1992), which was conducted prior to the EMDR, was partly lost. This is not uncommon in highly dissociative clients (Loewenstein et al., 2024). We then returned to phase 1 treatment and continued with our main treatment modalities, relational psychodynamics (Lemma et al., 2008), hypnosis including ego state interventions (Kluft, 2012; Watkins and Watkins, 1997), neurofeedback (Nicholson et al., 2020; van der Kolk et al., 2016) and AT. However, the pace of healing has been slow, and Clara still struggles to set healthy boundaries both at work and in close relationships.

Clara often became dysregulated in sessions. When her arousal responses became too overwhelming for her, they were followed by a plethora of dissociative symptoms. In sessions, Clara could momentarily switch to becoming numb, intellectualizing, and/or forgetting the moment that had just passed. This shift is a sign of increased cognitive control and inter-identity avoidance of trauma-related knowledge. Perhaps she used her CEN to not know about herself (Dimitrova et al., 2024) and overmodulated her subcortical brain activity (Lanius et al., 2010a; Lanius et al., 2010b; Lanius et al., 2018; Terpou et al., 2020). Through negative somatoform dissociation, e.g., not feeling the body (Nijenhuis et al., 1999), and psychoform dissociation (van der Hart et al., 2004), e.g., not noticing affects and having dissociative amnesia, she appeared to suppress somatic sensory information from reaching higher-order regions. This is consistent with the findings of Dimitrova et al. (2023) that emotional neglect is associated with dissociative amnesia. Like many sufferers of severe trauma-induced dissociation, she has had enormous problems calming or energizing herself. Although these symptoms have subsided, Clara still has problems being in touch with her emotions, affects, and body sensations. Her early life appeared to have left her with an incongruent, discontinuous, and disembodied experience of herself and her states of being.

Clara now attends sessions every 2 weeks. She experiences and describes a reduction of dreamlike states and depersonalization since DBR was added to the therapy. Moreover, she describes a more stable sense and connection with her body. According to the theory of DBR, we have identified and transformed the withdrawal reactions from early childhood and how these have affected her experience of herself.

**Figure 6:**
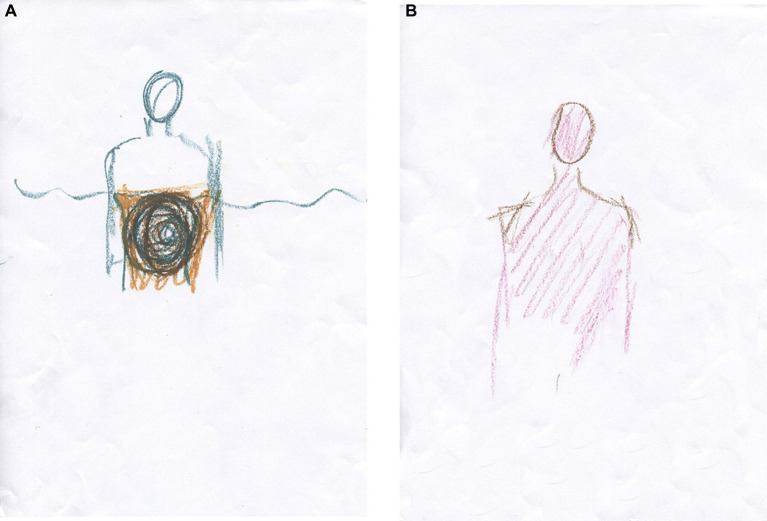
**(A)** Something that disturbs. One does not see what is going on underneath. **(B)** Between curiosity and fear, but a little more afraid. Afraid of being afraid. I get a little scared sometimes.

After 10 DBR sessions, Clara drew Figure 6A. Following an exercise designed to facilitate contact with the “proto-self” (Damasio, 1999; Corrigan et al., 2025), she made Figure 6B. The proto-self, as defined by Damasio (1999), refers to the areas in the brainstem that are involved in directing attention and perceiving the world. This concept is theorized to be the basic representation of the organism, arising from the brain’s constant interaction with the rest of the body. It is conceptualized as a coherent collection of neural patterns that map the organism’s physical structure, occurring moment by moment. In the context of DBR, addressing the proto-self is supposed to contribute to stabilizing the client.

Together, we shared and reflected on Clara’s courage to be more present again with her appraisals and embodied sense of feeling.

**Figure 7:**
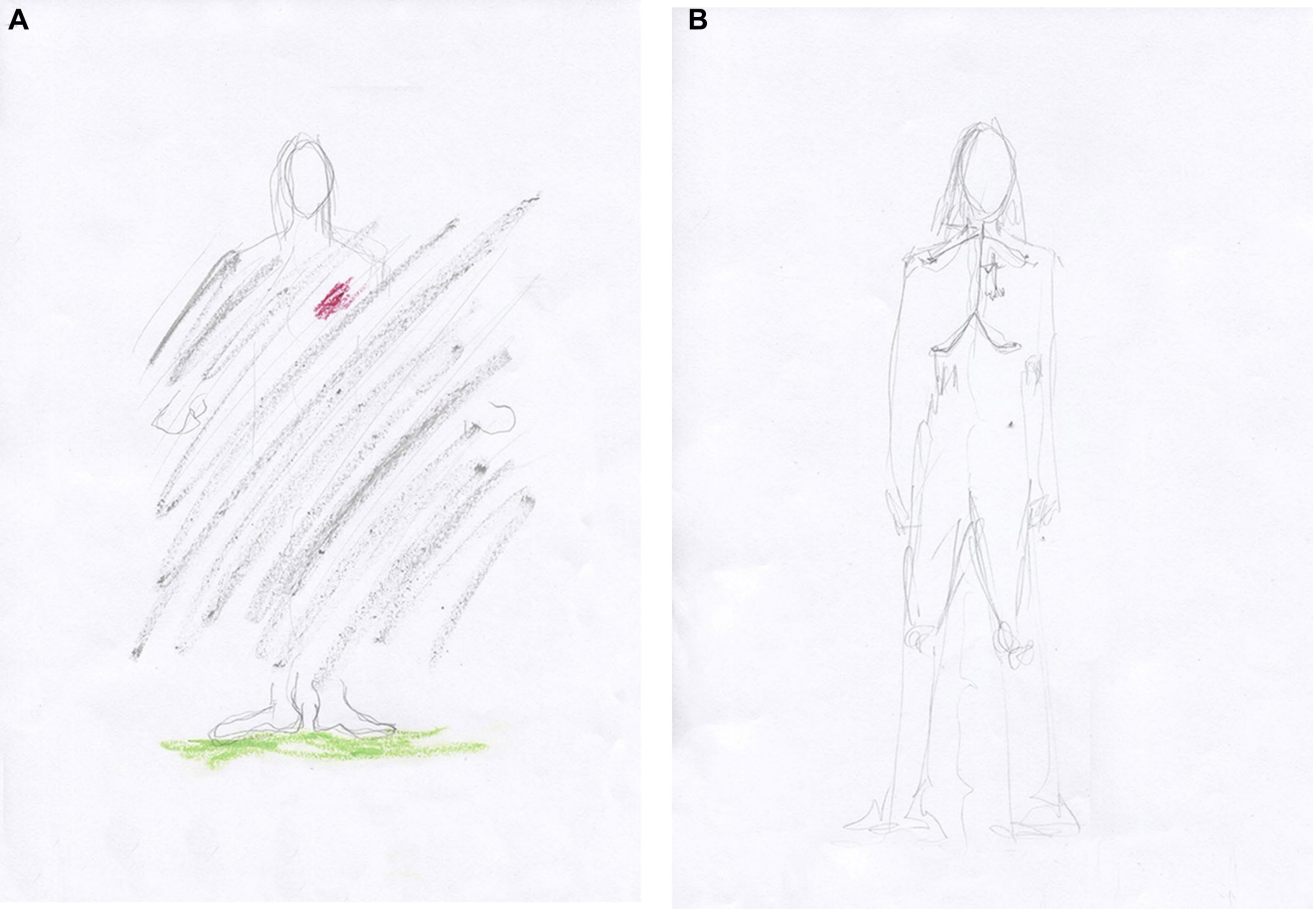
**(A)** Finding yourself. Now I have feet, hands, and a head. I have a heart and stand firm, but I am so diffuse inside. I cannot define myself. Gray is the boring thing in me—a void. I have no features because I do not know what I feel. **(B)** Being small when you are big. There are two smaller selves in me. My thighs are stronger—I can walk. I’m stable but feel strange, like Puck in A Midsummer Night’s Dream.

Half a year later, after 10 more DBR sessions, the client drew Figure 7A. “Finding yourself.” She was asked to investigate the gray in her drawing and make another drawing. She then drew a picture with thin pencil lines, Figure 7B, and called it “Being small when you are big.”

Two smaller selves can be seen in the picture. Relationally, the therapist needed to discover the smallest one, which, in many ways, was the real theme of this therapy. Clara was asked to make a new drawing, perhaps with oil pastels. At first, the drawing was light brownish with red wounds, and then most of the picture was covered in gray, possibly corresponding to the shame of not being recognized and held in a relationship (Schaverien, 1992). Furthermore, as signs in the art are ambiguous, the gray also provides information suggesting that Clara was on the verge of dissociating, using her well-trained ability to depersonalize when the experience became too overwhelming. Again, staying in the therapeutic relationship this time helped her not to dissociate, even though the pain depicted was beginning to overwhelm her. She called the image “hard to heal wounds.”

**Figure 8:**
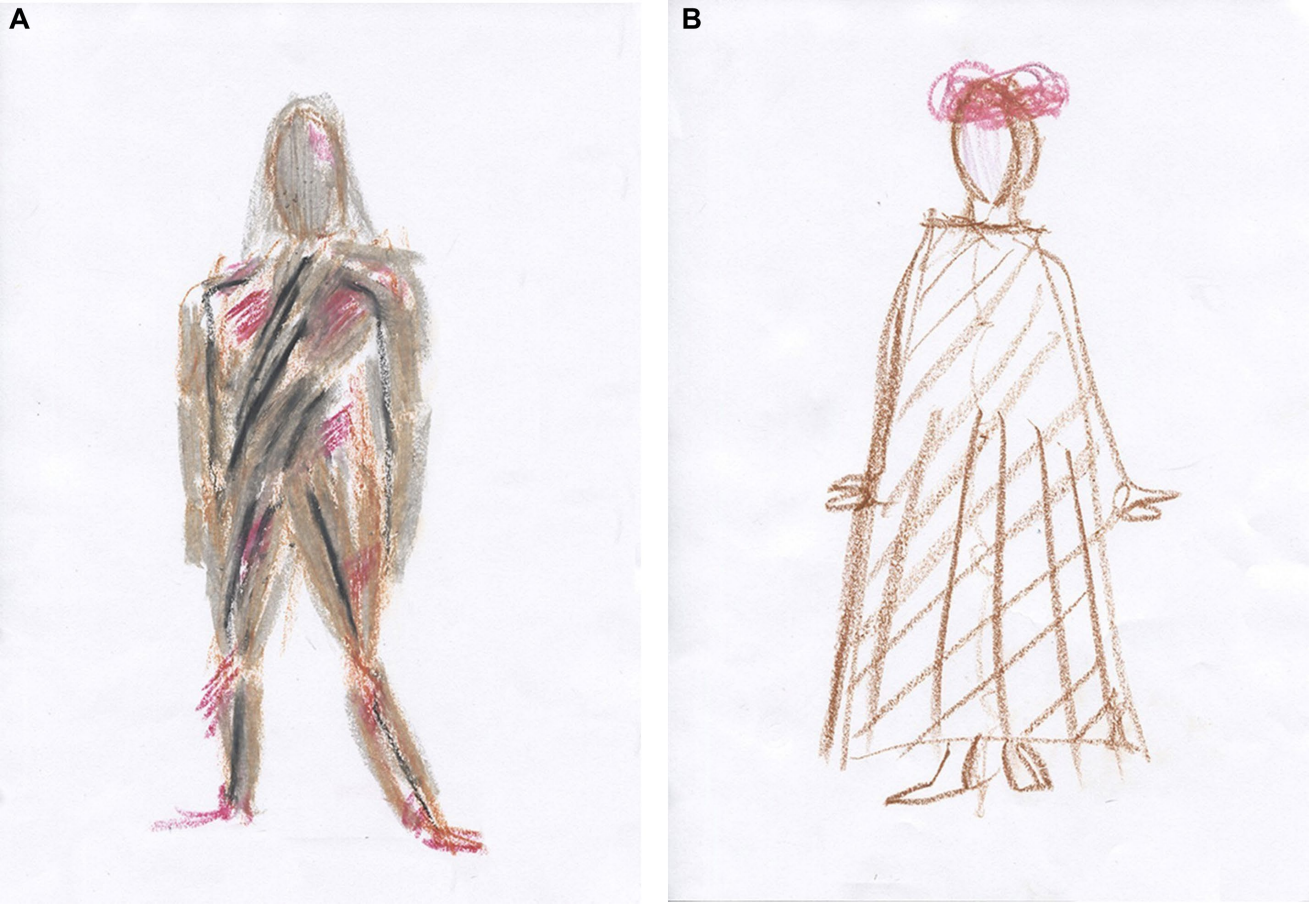
**(A)** Hard-to-heal wounds. No one ever listened. I can feel enormous guilt because others have suffered more, but I have not been cared for. No one cared about me, so when someone did care about me, I rejected it because I did not know what to do. **(B)** It is happier in the head. Now there are outlines (around the body).

Clara was relationally reassured and confirmed how lonely and painful it had been for her in childhood. Then, the theme in Figure 8A was used to activate the stimuli in DBR processing. She held on to her sensations but described wanting to run away from them “because it is almost unbearable.” She was able to stay present and allow the pre-affective shock to transform. Thereafter, waves of shame came and went within her. Just before finishing, she said, “It’s like my soul is being laid bare.” After the DBR, the client’s new perspective was: “Things are coming to the surface, and I cannot resist them. It’s a bit scary, but it comes so naturally. Now it’s a little more pink than gray (in me).” The client was then asked to make a final drawing, see Figure 8B.

Over the next 6 months, Clara had 12 more DBR sessions with the following themes: “No memories of attachment—just feeling so lonely. I thought I was worthless. When I’m rejected, I feel self-hatred and accept being maltreated. Getting something good feels forbidden, and I have no one to tell. My parents are dead, and they did not exist before—my fear of throwing myself off (my balcony). If I cannot perform, I’m worthless (and very lonely). That I’ve loved really mean guys. When I feel unworthy, I hit myself in the face (hard) and become like a little shameful child. That I come from the hard and loveless.”

**Figure 9:**
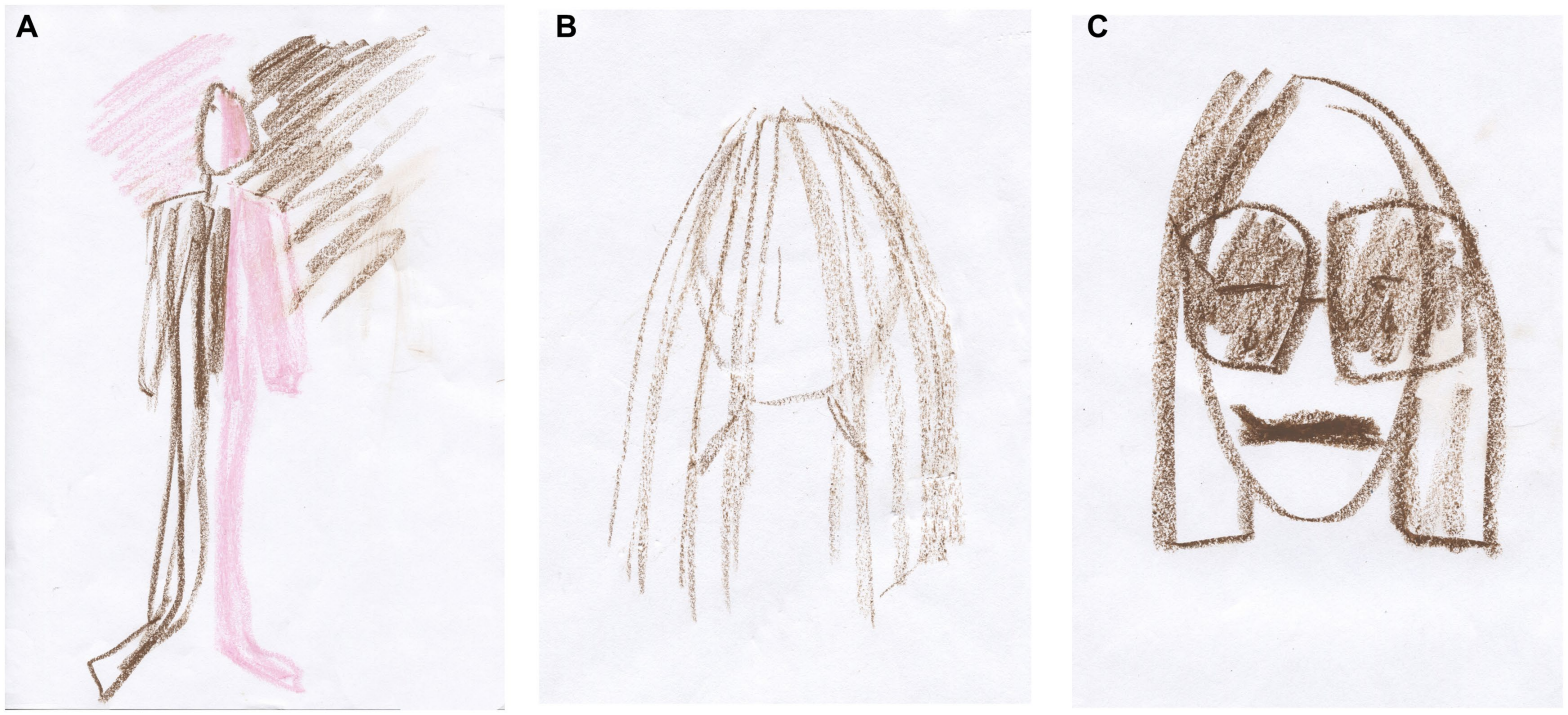
**(A)** The darkness in the light and the light in the darkness. **(B)** I want to hide something away; that I’m sad. **(C)** I’m about to open up to the world. There are tears behind the sunglasses.

In the last session shared in this vignette, Clara was asked to do pictures before and after the proto-self (Corrigan et al., 2025), followed by DBR processing and a final drawing. Regarding the first image, Figure 9A, Clara said, “The pink is faith in the future, but a dark shadow hangs over me all the time and is out of my control. It’s not pitch black, but it’s a struggle,” see Figure 9B.

In the proto-self grounding, Clara used her ability to care for loved ones as a resource. This reminded her of how little she was cared for as a child and she made Figure 9C.

The theme of the drawing was used as an activating stimulus in the DBR: “I want to hide that I’m sad because Mum and Dad did not care.” After releasing the pre-affective shock, Clara processed how ashamed she was as a child because her parents did not care, and she said, “and that’s where it ends,” and indicated that she had stopped feeling any new sensations. With the help of her therapist, she could go to the moment before she stopped being present with her sensations. Then, much emotional processing occurred, and Clara found her new perspective: “I am sad, and I can almost bear to mourn my childhood.” Finally, she made Figure 9C.

Before she left, Clara said, “Maybe I’m still the child inside that hurts so much.” The therapist replied, “Yes, maybe you are, until you can bear to feel what the child could not bear to feel, and you are starting to do that now.”

### Summary of the case vignette

In the case vignette, neither relational work nor neurofeedback, also a potentially recommended treatment modality for DID (Purcell et al., 2024), nor hypnosis appeared to transform the client’s dysregulation sufficiently. However, relational AT and DBR appeared to coincide with her heightened capacity to change. With this client, the interventions had to be tailored to her fragile integrative capacity, where she needed ongoing relational holding to dare to stay with her experiences. Such a strategy is often necessary with complexly traumatized and dissociative clients, as otherwise, their healing processes will plateau or stall. Such tailoring can be facilitated by increased knowledge of the human brain, innate effects, including shame, and our attachment system (Herman, 2011).

According to Loewenstein (2023), the treatment of DID aims at the continuity and accessibility of autobiographical memory, even if traumatic and shame-inducing. Improved access to and acceptance of the associated emotions and thoughts then follow, leading to clearer, more consistent reality testing and more deliberate and mindful behavior (Loewenstein, 2023). That appears to be happening now with Clara as her states of being become more integrated, and she experiences a more congruent self. Perhaps this is due to one of the methods, DBR or AT, or a combination of the methods, or perhaps it is because our previous work has brought us to where we are today. Now, after 15 years in therapy, she has reduced her impulses to throw herself off bridges and no longer feels lost. Her fear of flying, which brought her to therapy in the first place, has disappeared. She still struggles with the shame of having feelings and needs, although she describes that her fragile sense of her body is changing. She is experiencing more strength and happiness. She sets healthy boundaries more often.

#### Art therapy

Relationally attuned interventions on a psychodynamic basis were concretized in the relational art-making process. Then, challenging affects and states of being could be approached, expressed, and transformed. The embodied, here-and-now-based art potentially became a vehicle for change in the co-creative synchronicity of togetherness. The client’s drawings appeared to help her find themes to focus on. In addition, the fleeting quality of her implicit processes of change and the change achieved became explicit in her images.

#### DBR

Our work over the last 2 years has involved over 30 DBR sessions. DBR is hypothesized to address the regulatory nodes of the brainstem and midbrain. These regulate the higher limbic and cortical regions of the brain. DBR can be defined as a whole-mind intervention in the treatment of dissociation. The proto-self grounding (Corrigan et al., 2025) and the whole DBR intervention appeared to be helpful to the client. Perhaps DBR added a vehicle for transforming hitherto unbearable experiences of abandonment. These may have been stored as unbearable pain activation patterns in her midbrain and brainstem, often experienced as fleeting visceral sensations of threatening quality. Such pain is perhaps the deepest pain a human being can feel—that of the small child who is not helped to regulate the self and who is not held in a caring relationship.

#### The integrative approach

What Clara struggled with in sessions and life could be an effect of trauma-generated structural and functional changes in the cerebellum, affecting the connectivity of her brain’s macroscale networks. Such changes are common in early traumatization (Blithikioti et al., 2022). It is possible that these changes, if they were there, are now improving, perhaps through the combination of the integration offered by AT and DBR. AT was used to anchor her to the here and now, capture the implicit change processes, and find themes. The brain-informed method DBR was used to provide a gentle enough experience to allow a vulnerable DID client to process hitherto unbearable information about herself. No method exists without two-person psychology, a crucial approach when using methods with attachment-wounded clients. In relation to DBR, Clara said, “I feel that you are there, and I feel calm and that you are talking to me kindly. That is the most important thing for me. That, and that you do not think I am strange.”

Together with the drawings, DBR appeared to have anchored her existing capacity for integration. By helping her to bring her center of attention, the superior colliculus in the midbrain, online, she may have experienced reduced dissociative tendencies. Potentially, this gave her access to the capacity for change within her neurophenomenological self. In the attuned relational work in which AT and DBR were integrated, AT supposedly added that the client was “touched from within” (Bolwerk et al., 2014; Vaisvaser et al., 2024) when the anterior and posterior parts of her DMN were speculatively activated simultaneously. Reduced anxiety probably occurred through the ongoing activation of her orienting response, anchoring her in her senses. The agency she then experienced potentially activated enough joy and curiosity in the PAG of her midbrain. Overall, something in the therapy appeared to help her not to turn away from her ongoing experience of herself through depersonalization.

## Thematic analysis of the lived experience of being in therapy

The client was interviewed by the second author about having dissociation and being in psychotherapy, including DBR combined with AT. Of interest was the lived experience of being in therapy (Finlay, 2009). Qualitative researchers should strive to have their research informed by phenomenological concepts such as lifeworld, pre-reflective experience, and the lived body (Zahavi, 2019). The interview, grounded in a participatory worldview, aimed to bring theory and practice together. Such an orientation is based on action research (AR) (Lewin, 1946; Reason and Bradbury, 2008; Titchen, 2015), which aims to find practical solutions to problems that people care about. The method implies reflection in action, for example, being both situated and reflexive (Riley and Reason, 2015). New insights and knowledge can then be gained. The method can enable practitioners to explore their own practice.

Participatory action research (PAR) (Kemmis and Mctaggart, 2008; Purcell et al., 2024) aims to improve and develop collaborative practice. Three other DID clients were invited to reflect on the interview questions developed initially by the first author before they were used. The client was then invited to reflect on and modify the questions used in the previous interview. Finally, the interview was analyzed by the first and second authors, who synthesized the data. Three themes were found: *Before therapy—loneliness. In therapy—getting help,* and *Now—moving towards togetherness*, see Table 1.

**Table 1:** To have dissociation and being in psychotherapy.

Before therapy—loneliness	In therapy—getting help	Now—moving toward togetherness
Turned off	Difficult but interesting	I have changed, and I am a part of humanity.
Alone	Finding solutions	I am deciding more and more about my life.
Paralyzed	To have someone to think together with	I can feel emotions and set boundaries, and I am starting to realize that shame is unnecessary.
Felt separated from the world	EMDR took away the nightmares	I can think clearly and share who I am with others.
	Neurofeedback removed the filter, and I could start thinking	New doors are opening.
Felt that the earth will perish	Art makes it real, and it can be hard	DBR has made me softer, and I can be myself without needing to be perfect.
	The therapist provides reassurance, and I find peace in learning	I feel grief over my previous numbness regret not being fully present.
	DBR makes me grounded, calm, more myself, with less shame	I would not be alive today without the therapy.

Clara highlighted what is happening to her through therapy in these three sentences: “The shame has diminished. I do not feel alone in the universe. I can like and accept myself.” At the end of the interview, Clara made a drawing that summarized the therapy. She said, “I sometimes go into space and float up, but DBR keeps me grounded. DBR means that the sun’s rays can reach me, and I can feel that my feet are on the ground and that my back is straight. I used to be more hunched over. She called the drawing “finding the air of life.”

## Discussion

Treatment of highly dissociative clients aims to restore agency, reduce depersonalization, and empower people to live their chosen lives. To achieve this, DID clients might benefit from relationally held integrative sensorimotor anchoring, exemplified by AT, combined with a “brain informed” treatment modality, which DBR exemplifies. This could result in their visceral sensations no longer haunting them as decontextualized bodily sensations. These changes may correspond to the decoupling of hyperconnectivity between the PAG and SMN, the CEN and DMN, and the posterior DMN and SMN. Potentially, if the connectivity between the anterior and posterior nodes of their DMN is re-established, they may be able to mentally “time travel” and remember the past with the freedom to redirect attention to the present as and when they wish (Kearney and Lanius, 2024).

Hypothetically, therapy could alter the brain functions and connectivity patterns of highly dissociative clients to resemble those of healthy controls. Potentially, this can lead to those patterns of neural activity within their primary sensorimotor circuits becoming multimodal representations in their association cortices. The individuals can be safely embodied—here and now—and their frozen memories (Herman, 1992) will no longer be trapped in frozen memory systems. As a result, the frozen carriers of intrusions and flashbacks may be transformed into conscious agents capable of revising their autobiographical narratives.

Such changes may speculatively begin at the brainstem level (Corrigan and Christie-Sands, 2020) and target the brain’s functional networks (Lotfinia et al., 2020; Purcell et al., 2024). Potentially, trauma and brain-informed therapy methods are needed to access the early turned-away brains of attachment-wounded DID clients (Purcell et al., 2024). There may then be increased activation of brain circuits that reduce depersonalization. Otherwise, when disconnected from bodily sensations, people report a loss of connection to their sense of self and personal history (Simeon and Abugel, 2006). Ferroni et al. (2024) suggest that the ability to orient oneself flexibly through time and project oneself into the future may be associated with an intact core sense of self and reduced depersonalization. From such a perspective, therapeutic interventions targeting reduced depersonalization are called for.

The changes in the case vignette could hypothetically correlate with changes in the client’s functional networks. Brain scans would be needed to check whether her overmodulation of subcortical brain activity through her CEN has begun to change, whether her SMN and posterior DMN are in the process of decoupling, and whether the functional connectivity of her anterior and posterior DMN has begun to normalize. The likely neurophysiological basis for her reduced number of episodes of depersonalization in everyday life remains unexplored. Brain scans before, during, and after sessions would be required to verify such interpretations. However, to a lesser extent, the client appears to be suppressing her somatic sensory information from reaching higher-order regions.

Speculatively, the changes in presumed network connectivity and increased experience of agency in psychotherapy outlined above could lead to lasting neuroanatomical changes. These could hypothetically include a regained function and volume of the flocculus in the deep cerebellum, leading to an experience of a more coherent and embodied self. Regarding the amygdala and related structures, an RCT of prolonged exposure as a treatment for PTSD clients found greater reductions in amygdala-frontal and insula-parietal connectivity associated with greater reductions in PTSD symptoms (Fonzo et al., 2021). According to Manthey et al. (2021), successful psychological treatments for PTSD could potentially work via upregulation of the medial PFC, which may thus be involved in symptom reduction. Whether such changes occurred with this treatment has not been investigated. There are also no studies of treatments for DID to support such hypotheses. However, when clients are relationally regulated, exteroceptive and interoceptive sensory input is modulated. Clients can then use their embodied cognitive capacities, and their interoceptive triggers can also be relieved.

## Conclusion

With great respect for the methods that have been shown to be valuable in treatment through RCTs and individual cases, art therapists working with severe trauma should tailor their interventions to the neurophysiology of the traumatized brain. From such a perspective, calming, regulating, and transforming RAT interventions are thought to reach specific areas of the deep brain and enhance favorable coupling and uncoupling of the brain’s functional networks. Pictorial artifacts can be both vehicles for therapeutic change processes and temporary endpoints or proxy measures in terms of states and functions rather than traits, with a certain perspective on experienced safety and regained existential health (Gerge, 2018b,c; Payano Sosa et al., 2023). RAT provides a vehicle for “being with” while the client’s epistemic trust changes and deepens. When working with severe dissociation, non-verbal arts-based psychotherapeutic approaches can bridge the dissociation (International Society for the Study of Trauma and Dissociation, 2011; Loewenstein, 2023).

DBR offers a valuable hypothesis about the processes of appraisal and dissociation at a whole-brain level, including the midbrain and brainstem as regulative centers of attention and arousal. By incorporating the hypotheses of Corrigan and Christie-Sands (2020), Corrigan et al. (2025), and Kearney and Lanius (2022, 2024), (art) therapists can enhance their ability to heal attachment deficits, post-traumatic conditions, and dissociation. Supposedly, because the deep brain appears to regulate much of the higher cortical functions (Blithikioti et al., 2022; Kearney and Lanius, 2022; Panksepp, 2012) and sense of self (Panksepp and Northoff, 2009)—two capacities that are globally impaired in trauma-related disorders.

According to the important findings of Lanius et al. (2020), therapists need to know what kind of clients they are sitting with. This can be assessed in part through verbal or written assessments and clinical interviews, where several diagnostic tools for DID are valid and recommended (Loewenstein et al., 2024).

## Limitations

One limitation is that the first author carried out the clinical intervention presented in the case vignette; therefore, the second author conducted an interview to give voice to the client. Another limitation is that the mechanisms explaining the value of art therapy are unknown and remain to be uncovered (King and Chatterjee, 2024), and the value of DBR in treating severe dissociation is currently only hypothetical. Another problem is the limited evidence due to the paucity of quantitative research studies on the efficacy of AT, RAT, and DBR in treating the psychobiological syndrome of DID (Purcell et al., 2024). Although consistent with recent neurobiological and neurophysiological findings, the clinical vignette’s therapeutic claims are not supported by brain scans. This makes the causality of the potential changes in the clinical vignette speculative, to say the least. Therefore, the potential value of the combination of RAT and DBR is preliminary at best.

### Further development

An understanding of the neurobiology of dissociation and DID is necessary for the development of psychotherapeutic interventions for DID. Purcell et al. (2024) emphasize that the current state of DID is similar to the state of PTSD in the 1970s—highly stigmatized, misunderstood, underfunded, and understudied. They call for further advances in the neurobiological understanding of dissociation and DID that can inform the development of novel psychotherapeutic interventions. DBR and clinical hypnosis are two promising examples. Whether RAT could be considered a neuroscientifically based psychotherapeutic intervention for severe dissociation needs to be established by quantitative research and neuroimaging techniques that capture changes in brain function before and after therapy. This could provide more conclusive evidence of the potential neurophysiological effects of RAT and other methods.

Both RAT and DBR in the treatment of well-diagnosed DID clients, for diagnostic recommendations, see Loewenstein et al. (2024), need to be evaluated as separate treatment modalities with initial pilot studies. If successful, controlled trials with waiting list controls and comparisons to standard treatments should be conducted. A major challenge is that treating severe dissociative disorders like DID typically takes years. This makes the randomized controlled trial (RCT), the gold standard for outcome research, not an optimal lens for studying treatments for DID, as the disorder is not amenable to brief interventions (Brand et al., 2009; Nijenhuis, 2017). However, some steps, such as the introduction of DBR or another method, can be analyzed sequentially in the shorter term. Further research should include a small exploratory study of the usefulness of AT and DBR in combination in working with highly dissociative clients, and a therapy manual could also be developed.

## Data Availability

The datasets presented in this article are not readily available because the clinical material cited is not a public document. Requests to access the datasets should be directed to Anna Gerge, anna@insidan.se.
